# The H3.3K27M oncohistone affects replication stress outcome and provokes genomic instability in pediatric glioma

**DOI:** 10.1371/journal.pgen.1009868

**Published:** 2021-11-09

**Authors:** Irena Bočkaj, Tosca E. I. Martini, Eduardo S. de Camargo Magalhães, Petra L. Bakker, Tiny G. J. Meeuwsen-de Boer, Inna Armandari, Saskia L. Meuleman, Marin T. Mondria, Colin Stok, Yannick P. Kok, Bjorn Bakker, René Wardenaar, Jonas Seiler, Mathilde J. C. Broekhuis, Hilda van den Bos, Diana C. J. Spierings, Femke C. A. Ringnalda, Hans Clevers, Ulrich Schüller, Marcel A. T. M. van Vugt, Floris Foijer, Sophia W. M. Bruggeman

**Affiliations:** 1 Department of Ageing Biology/ERIBA, University of Groningen, University Medical Center Groningen, Groningen, the Netherlands; 2 Glial Cell Biology Laboratory, Biomedical Sciences Institute, Federal University of Rio de Janeiro, Rio de Janeiro, Brazil; 3 Department of Pathology and Medical Biology, University of Groningen, University Medical Center Groningen, Groningen, the Netherlands; 4 Department of Histology and Cell Biology, Faculty of Medicine, Public Health and Nursing, Universitas Gadjah Mada, Yogyakarta, Indonesia; 5 Department of Medical Oncology, University of Groningen, University Medical Center Groningen, Groningen, the Netherlands; 6 iPSC/CRISPR facility, Department of Ageing Biology/ERIBA, University of Groningen, University Medical Center Groningen, Groningen, the Netherlands; 7 Princess Máxima Center for Pediatric Oncology, Oncode Institute, University Medical Center Utrecht, Utrecht, the Netherlands; 8 Hubrecht Institute, Royal Netherlands Academy of Arts and Sciences (KNAW), Oncode Institute, University Medical Center Utrecht, Utrecht, the Netherlands; 9 Research Institute Children’s Cancer Center Hamburg, Hamburg, Germany; 10 Department of Pediatric Hematology and Oncology, University Medical Center Hamburg-Eppendorf, Hamburg, Germany; 11 Institute of Neuropathology, University Medical Center Hamburg-Eppendorf, Hamburg, Germany; Hopp-Children’s Cancer Center KiTZ and German Cancer Research Center DKFZ, GERMANY

## Abstract

While comprehensive molecular profiling of histone H3.3 mutant pediatric high-grade glioma has revealed extensive dysregulation of the chromatin landscape, the exact mechanisms driving tumor formation remain poorly understood. Since H3.3 mutant gliomas also exhibit high levels of copy number alterations, we set out to address if the H3.3K27M oncohistone leads to destabilization of the genome. Hereto, we established a cell culture model allowing inducible H3.3K27M expression and observed an increase in mitotic abnormalities. We also found enhanced interaction of DNA replication factors with H3.3K27M during mitosis, indicating replication defects. Further functional analyses revealed increased genomic instability upon replication stress, as represented by mitotic bulky and ultrafine DNA bridges. This co-occurred with suboptimal 53BP1 nuclear body formation after mitosis *in vitro*, and in human glioma. Finally, we observed a decrease in ultrafine DNA bridges following deletion of the K27M mutant *H3F3A* allele in primary high-grade glioma cells. Together, our data uncover a role for H3.3 in DNA replication under stress conditions that is altered by the K27M mutation, promoting genomic instability and potentially glioma development.

## Author summary

The childhood brain cancer high-grade glioma is a devastating disease that almost invariably ends in the death of the patient. The discovery that mutations in histone H3 variants are predominant in this type of cancer created great interest into the mechanism of transformation of these so-called oncohistones. Yet whereas most research is directed at understanding the role of mutant H3 in deregulating gene expression, we studied if and how it contributes to genomic instability–a research avenue that remains mostly unexplored. We discovered that the H3.3K27M oncohistone increases sensitivity to replication stress during S-phase, ultimately leading to mitotic aberrancies. This can explain why childhood high-grade glioma is among the most genomically unstable cancers.

## Introduction

Pediatric high-grade glioma (HGG) is a common childhood brain malignancy for which no adequate treatment exists [1]. The discovery of mutant histones as most frequently occurring genetic alteration in these cancers provided a novel angle for developing anti-cancer therapy, and generated broad interest into these ‘oncohistones’ [1–3]. So far, five different histone mutants (H3.1K27M, H3.2K27M, H3.3K27M, and H3.3G34R/V) have been identified that target either Lysine 27 or Glycine 34 on one of the three histone H3 variants. Interestingly, pediatric HGG carrying different histone mutations have a distinct age of onset and harbor different anatomical, clinical and molecular features, suggesting that each mutant has unique oncogenic characteristics [4–6]. Several functional studies have confirmed the tumor-driving capacity of the oncohistones and revealed their impact on chromatin modifying proteins, chromatin composition and gene expression [7–14]. This is in line with the apparent selective pressure to mutate residues that either are, or flank epigenetic modification sites involved in gene regulation.

However, it remains elusive how these transcriptional changes lead to tumor formation [15]. Therefore, it is possible that (onco)histone functions unrelated to gene regulation contribute to tumorigenesis as well. In this respect, it is intriguing that pediatric HGG with mutations in the non-canonical histone H3.3 exhibit particularly high rates of genomic and chromosomal instability (GIN/CIN) [16]. This raises the question whether the H3.3K27M mutation can promote GIN/CIN, as has been suggested for H3.3G34R/V, which would contribute to intra-tumor heterogeneity and tumor aggressiveness [17–22]. Indeed, in addition to its canonical role in the packaging of chromatin and modulation of gene expression, histone H3.3 carries out functions linked to maintenance of genomic integrity. For instance, H3.3 is enriched in regions where genome stability is at risk, such as telomeres and pericentric heterochromatin. Further, its loss leads to mitotic defects in mouse embryonic stem cells, suggesting a role for H3.3 in chromosomal stability. In addition, H3.3 has been implicated in both single- and double-strand DNA break repair and H3.3 mutant cells have increased sensitivity to DNA damage [23–27]. Intriguingly, unlike H3.1 mutant HGG, which mostly harbors mutations in developmental pathways, mutations in H3.3 mutant HGG are enriched for genes involved in DNA damage repair pathways [4,28].

Since unrepaired DNA damage ultimately leads to CIN/GIN, we hypothesized that H3.3K27M might establish a permissive background for genome instability. In the developing brain, genomic integrity is mostly threatened by highly prevalent replication stress-dependent DNA lesions [29–31]. Hence, we focused on how H3.3K27M affects the response to replication stress. We found that H3.3K27M increases sensitivity to replication stress, leading to mitotic abnormalities including DNA ultrafine bridges. These observations could explain the ongoing genomic and chromosomal instability that we identified in pediatric high-grade glioma.

## Results

### Histone H3.3^K27M^ mutant cells exhibit mitotic abnormalities

**Fig 1:**
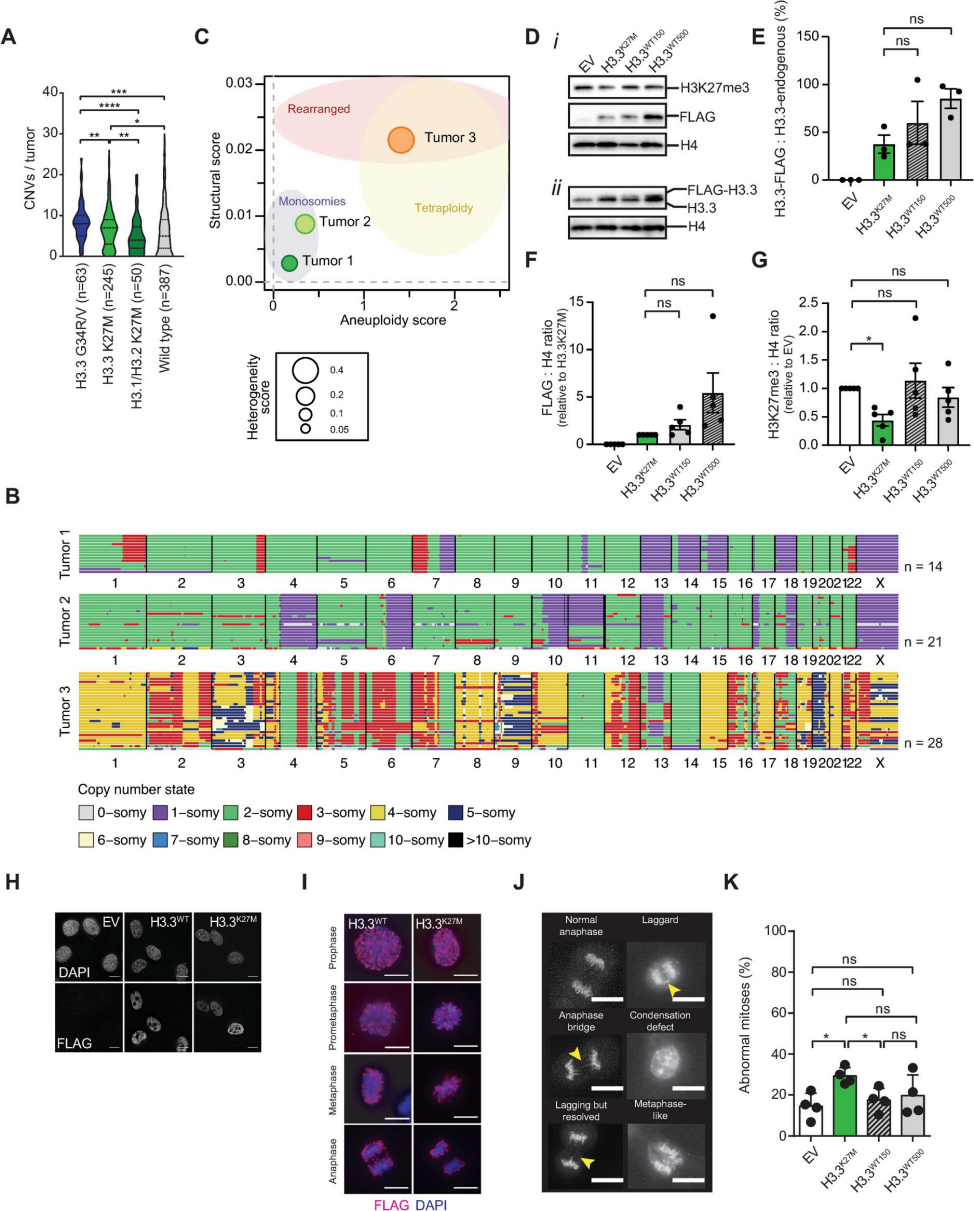
The H3.3K27M oncohistone is associated with aneuploidy, intratumor heterogeneity, and genomic and chromosomal instability. **(A)** Distribution of chromosome copy number variations (CNVs) across a set of pediatric high-grade gliomas (HGG). Data are represented as medians (— — —) and quartiles (- - - -); *P = 0.0289, **P = 0.0064 and P = 0.0066, ***P = 0.002,****P≤0.0001 (Mann Whitney non-parametric t-test). Source data for this panel were derived from Mackay et al, Cancer Cell 2017, and re-used with permission [4]. **(B)** Single-cell Whole Genome Sequencing data for n = 14 (tumor 1), n = 21 (tumor 2) and n = 28 (tumor 3) cells from human H3.3K27M mutant pediatric HGG biopsies. Each line represents the karyotype of a single cell, organized by chromosome number. Colors represent ploidy. **(C)** Structure, aneuploidy and heterogeneity plot of n = 3 pediatric HGG tumors. The structural score (plotted on the Y-axis) represents the number of structural abnormalities observed in the tumor, as it quantifies the frequency of copy number changes within chromosomes. The aneuploidy score (plotted on the X-axis) quantifies the frequency of large copy number changes (whole chromosome or chromosome arms gains or losses). The heterogeneity score, represented by circle size, measures the extent of structural and numerical variation between the cells from one tumor. **(D)** Western blots depicting *i)* H3K27me3 and FLAG-H3.3 expression in H3.3^WT^, H3.3^K27M^ and EV control cells, and *ii)* Flag-H3.3 and endogenous H3.3 expression after 48 hrs of Doxycycline treatment. H4, histone H4, loading control. **(E)** Chart representing the percentage of FLAG-H3.3 expression compared to endogenous H3.3 in H3.3^WT^, H3.3^K27M^ and EV control cells determined by H3.3 antibody staining, as quantified by densitometry (paired one-way ANOVA, Bonferroni correction for multiple comparisons). **(F)** Chart representing relative FLAG-H3.3 expression in H3.3^WT^, H3.3^K27M^ and EV control cells determined by FLAG antibody staining and normalized to H4, as quantified by densitometry (paired one-way ANOVA, Bonferroni correction for multiple comparisons). **(G)** Chart representing the relative H3K27me3 expression compared to H4 in H3.3^WT^, H3.3^K27M^ and EV control cells relative to EV, determined by H3K27me3 antibody staining, as quantified by densitometry. *P = 0.0128 (paired one-way ANOVA, Dunnett correction for multiple comparisons). **(H)** Immunofluorescent images showing cellular localisation of FLAG-H3.3 at interphase, and **(I)** at all stages of mitosis. Scale bars represent 10 μm. **(J)** Images showing the most common mitotic features that were scored for in time lapse experiments. Scale bar represents 10 μm. Arrows indicate mitotic defects. **(K)** H3.3^K27M^, H3.3^WT150^, H3.3^WT500^ and EV control cells were treated for 48 hrs with Doxycycline and time lapse imaged for 16 hrs. Mitotic features were scored as normal or abnormal. Data represent mean percentages of abnormal mitoses ± SD (n = 4 experiments), with a minimum of 20 mitoses per condition. *P = 0.0109 and 0.0331 (one-way ANOVA).

To explore the GIN/CIN phenotype of histone mutant pediatric glioma and quantify the extent of copy number alterations, we analyzed DNA copy number variations (CNVs) in n = 745 pediatric HGG using a previously published dataset [4]. We found that histone H3.3 mutant gliomas have a greater degree of CNVs compared to histone H3.1 mutant gliomas and H3 wild type gliomas (Fig 1A and S1 Table), with H3.3G34R/V HGG being more aneuploid than H3.3K27M HGG. Since H3.3G34R/V and H3.1K27M have previously been associated with genomic instability in functional studies, we focused our research on H3.3K27M HGG, the most prevalent type of H3 mutant glioma [21,32].

It is currently not well-understood whether CNVs are typically clonal or heterogeneous within H3.3K27M HGG, the latter being a sign for ongoing GIN/CIN [33]. This is relevant, as ongoing GIN/CIN promotes karyotype evolution and is associated with acquired drug resistance and a poor patient prognosis [34]. Because intratumor karyotype heterogeneity is a good indicator of ongoing GIN/CIN, we next performed shallow single-cell whole genome sequencing for three pediatric H3.3K27M HGGs to quantify this (Fig 1B) [35]. All three tumors displayed high grade aneuploidy, with tumor 1 and 2 showing mostly monosomies and tumor 3 showing mostly tetrasomies. Possibly, tumor 3 underwent a whole genome duplication event as most chromosomes are tetraploid with some chromosomes appearing lost compared to a tetraploid state (Fig 1B). All three tumors displayed whole chromosome copy number changes as well as structural copy number changes, in agreement with previous studies [4]. Furthermore, we observed recurrent abnormalities between the three tumors that included gain of chromosome 7p and loss of 7q (tumors 1 and 3), as well as loss of chromosomes 13 (tumors 1–3) and 14 (tumors 1 and 3), all of which have been described before as recurrent lesions in pediatric HGG (Fig 1B) [4]. Note that for tumor 3, because of the whole genome duplication event, trisomies and disomies are considered losses compared to a tetraploid precursor.

Importantly, when assessing the karyotype landscape of individual tumors, we also observed chromosome (fragment) gains and losses that were unique to individual tumor cells, indicating intratumor karyotype heterogeneity and thereby, ongoing GIN/CIN. To quantify this further, we calculated the aneuploidy, structural and intratumor heterogeneity scores. This revealed that tumor 3 displayed the highest aneuploidy, structural and heterogeneity scores, while tumor 1 displayed the lowest scores (Fig 1C). Taken together, these observations confirm that H3.3K27M HGG is a highly aneuploid cancer with recurrent chromosomal abnormalities. They further indicate that H3.3K27M HGGs suffer from ongoing GIN and CIN with relatively high rates that vary between tumors.

This prompted us to further investigate a role for H3.3K27M in the maintenance of genomic integrity. Hereto, we generated a cell culture model system that allowed us to study genomic maintenance in a non-transformed background, and thus identify primary functions of (K27M mutant) histone H3.3 (Fig 1D–1H). We took advantage of the human cell line hTert-RPE1 (hereafter referred to as RPE1), which is widely used in chromosomal instability and aneuploidy studies due to its well-defined stable karyotype [36,37]. Three different cell lines were generated: RPE1 cells containing Doxycycline-inducible FLAG-tagged wild-type histone H3.3 (further referred to as H3.3^WT^) to account for phenotypes due to mild histone overexpression [38]; RPE1 containing FLAG-tagged K27M mutant histone H3.3 to identify mutation-specific phenotypes (H3.3^K27M^); and an empty vector control cell line (EV) (Fig 1D–1H). Importantly, the Doxycycline-inducible system allowed us to tightly control timing and levels of overexpression, which is essential since only 4 to 18% of total histone H3 is mutated in HGG, and because we wished to investigate early events following (mutant) H3.3 expression [10]. Thus, we opted for subtle over-expression of FLAG-tagged H3.3 compared to endogenous H3.3 for all experiments, with FLAG-H3.3 never exceeding endogenous H3.3 protein levels (Fig 1D and 1E). Further, H3.3WT overexpression levels were equilibrated to H3.3K27M by also treating H3.3^WT^ cells with a lower Doxycycline concentration (150 ng/ml versus 500 ng/ml for H3.3^WT^), such that H3.3K27M expression would not be higher than in H3.3^WT150^ (Fig 1D and 1F). We also performed all experiments within 2–3 days after FLAG-H3.3 expression induction to isolate primary phenotypes (unless stated otherwise). We validated our model by showing a modest reduction of the H3K27 tri-methylation (H3K27me3) mark in H3.3^K27M^ cells after 2 days of FLAG-histone induction, as has been reported previously [10,39] (Fig 1D and 1G). We also confirmed that FLAG-H3.3 expression was predominantly in the nucleus (Fig 1H).

We then asked if there is a relationship between H3.3K27M expression and chromosomal instability. First, we verified that H3.3WT and H3.3K27M were similarly distributed on condensed chromatin during all stages of mitosis (Fig 1I). To visualize real-time chromosome behavior during mitosis, we performed time lapse imaging and quantified mitotic timing and abnormalities (Figs 1J and 1K, and S1). We observed mitotic abnormalities in approximately twenty percent of mitotic H3.3^WT150-500^ cells, as previously described by others [38]. However, the H3.3 K27M mutation increased the rate of abnormal mitoses, which was significantly different from H3.3^WT150^ cells, suggesting a possible role for the H3.3^K27M^ oncohistone in chromosomal instability (Fig 1K).

To understand the origin of the observed increase in CIN, we tested whether H3.3K27M oncohistone expression attenuates the spindle assembly checkpoint (SAC), a key mitotic fail-safe mechanism that ensures faithful chromosome segregation in mitosis (reviewed in [40]). Hereto, we challenged Doxycycline-induced cells with Nocodazole, a microtubule poison that arrests cells in mitosis if the SAC is fully functional (S1A Fig). Flow cytometry analysis did not show a difference in the ability of either H3.3^WT^, H3.3^K27M^, or EV control cells to arrest in mitosis as measured by mitotic H3S10-phosphorylation (S1B Fig, upper panel). Other mitotic phosphorylation histone marks, such as serine 28 (H3S28P) and the H3.3 specific Serine 31 (H3.3S31P) that are in the proximity of the mutated Lysine 27, were also unchanged (S1B Fig, middle and lower panels, respectively). Furthermore, time lapse imaging showed comparable mitotic timing in all three cell lines (S1C Fig). Altogether, this suggests adequate mitotic progression and a functional SAC in H3.3^K27M^ cells that fail to explain the increased rate of abnormal mitoses (Fig 1K).

### The histone H3.3K27M mutant has an altered protein interaction network in mitosis

**Fig 2:**
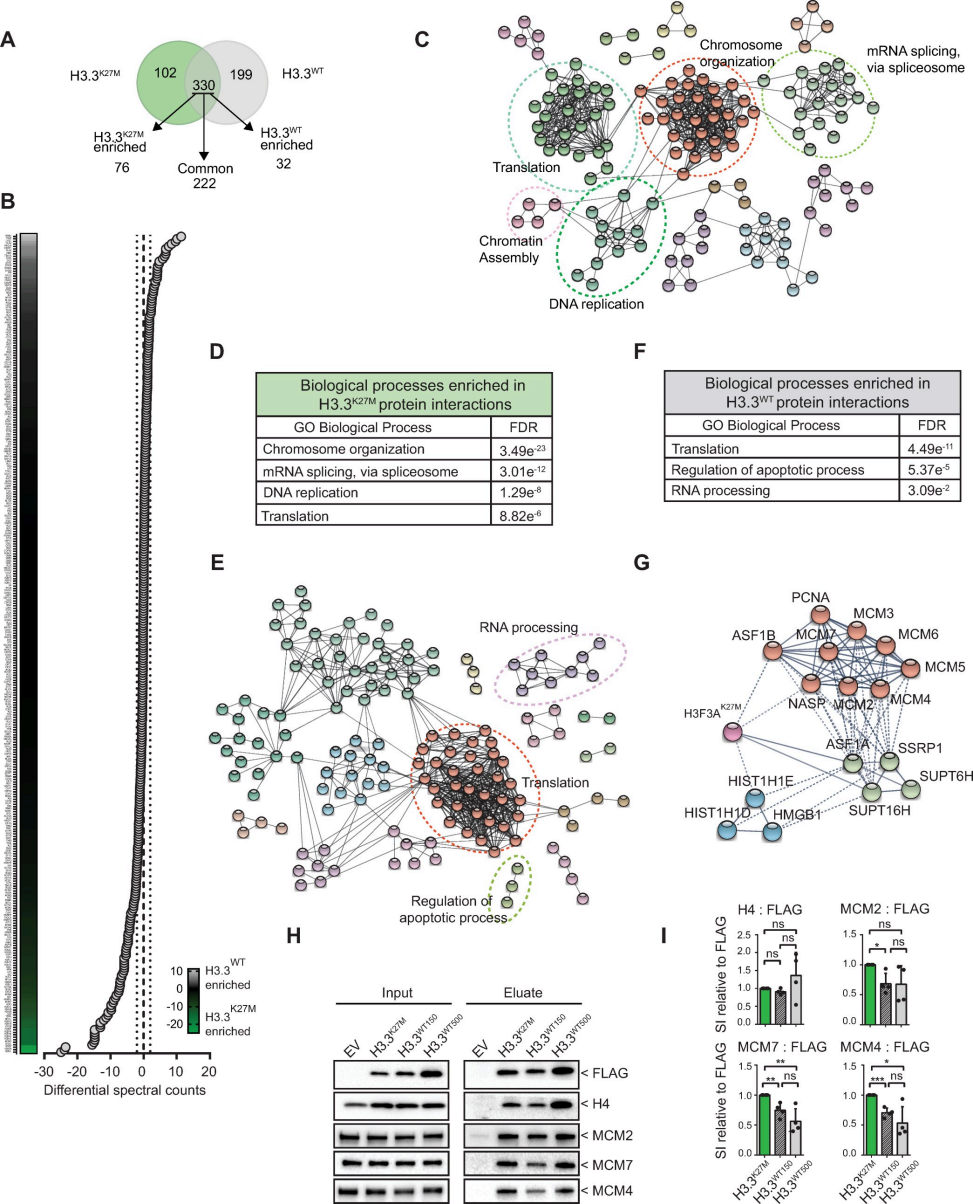
The Histone H3.3^K27M^ mutant has an altered mitotic protein interaction network. **(A)** Venn diagram showing the distribution of FLAG-histone interacting proteins that are either specific for H3.3^WT^ (n = 199) and mutant H3.3^K27M^ (n = 102) cells, or common/enriched (n = 330) as identified by LC-MS/MS. **(B)** Graph depicting semi-quantitative spectral counts of H3.3^WT^ and H3.3^K27M^ common binding partners (black, n = 222) (differential spectral counts between dashed lines), and enriched binding partners (green: H3.3^K27M^ - enriched, n = 76; grey: H3.3^WT^—enriched, n = 32). **(C)** H3.3^K27M^ specific/enriched interaction network for Biological processes as analyzed using STRING. The illustrated network map was simplified for clarity by manual curation. Dashed circles represent a group of interacting proteins belonging to one Biological process. **(D)** GO-term analysis (for Biological processes) of interacting proteins using STRING reveals significant enrichment for chromosome organisation, mRNA splicing, DNA replication and translation in enriched H3.3^K27M^ binding partners. GO = gene ontology, FDR = false discovery rate. **(E)** H3.3^WT^ specific/enriched interaction network as analyzed using STRING. The illustrated network map was simplified for clarity by manual curation. Dashed circles represent a group of interacting proteins belonging to one biological process. **(F)** GO-term analysis (for Biological processes) of interacting proteins using STRING reveals significant enrichments for translation, regulation of apoptotic process and RNA processing in enriched H3.3^WT^ binding partners. GO = gene ontology, FDR = false discovery rate. **(G)** DNA replication enriched network showing binding partners relation to the histone mutant H3.3K27M. **(H)** Western blot validation of FLAG-immunoprecipitations in H3.3^WT^, H3.3^K27M^ and control mitotic lysates. **(I)** Quantification of the histone-bound fraction of H4, MCM2, 7 and 4 by densitometry. Signal was normalized to FLAG, and H4 was used as positive control. Data are presented as means ±SD (n = 4 experiments). *P = 0.0138 and 0.0107; **P = 0.0054 and 0.0057; ***P = 0.0003 (Multiple t-tests).

In mitosis, the chromatin undergoes significant changes and histones become the substrates and scaffolds for a myriad of proteins to allow proper DNA segregation [41–43]. Therefore, we next set out to investigate the protein-histone H3.3(K27M) interactions during mitosis to further decipher the cause of the observed genomic instability. For this purpose, a FLAG-Immunoprecipitation (FLAG-IP) was performed on EV, H3.3^WT^, and H3.3^K27M^ mitotic nuclear extracts (S2A Fig). Flow cytometry analysis confirmed that our synchronization and shake-off protocol efficiently enriched the input fractions for mitotic H3S10P positive cells (S2A and S2B Fig). Then, mass-spectrometry analysis was performed on the eluted protein fractions to identify H3.3 interactors (Figs 2 and S2 and S2–S5 Tables). In total, around 750 proteins were identified in each cell line (S2 Table). Following background subtraction and normalization, we obtained curated binding partner lists in which we identified n = 199 (H3.3^WT^) and n = 102 (H3.3^K27M^) unique interactions, and n = 330 common interactions (Fig 2A). Within the common interactors, n = 32 (H3.3^WT^) and n = 76 (H3.3^K27M^) interactors were enriched for either H3.3^WT^ or H3.3^K27M^. Fig 2B graphically represents the common (not enriched), and enriched interactors.

To identify deregulated biological processes in mitotic H3.3^K27M^ cells, we grouped interactors as either common (not enriched, n = 222), or unique plus enriched (for either H3.3^WT^, n = 231; or H3.3^K27M^, n = 178), and performed GO-term analysis (STRING). As expected, common interactors showed enrichment for processes related to chromosome organization, with chaperones (*e*.*g*., DAXX), histones (*e*.*g*., H2A, H2B, H4) and chromatin readers and modifiers (*e*.*g*., CBX3) constituting the main hits (S2C and S2D Fig and S3 Table). We also found enrichment for mRNA splicing via spliceosome (HNRNPR, HNRNPK, HNRNPC) and translation machinery, including ribosome components (RPL and RPS subunits) and translation initiation factors (EIF3s and EIF4s) in line with earlier findings [44] (S2C and S2D Fig and S3 Table).

We subsequently analyzed the H3.3^WT^ and H3.3^K27M^ unique/enriched interactions (Fig 2C–2F and S4 and S5 Tables). Both H3.3^K27M^ (Fig 2C and 2D) and H3.3^WT^ (Fig 2E and 2F) showed enrichment for processes related to translation, albeit through different subsets of RPS and RPL variants (Fig 2C–2F and S4 and S5 Tables). Additionally, we observed enrichment for RNA processing in H3.3^WT^, and mRNA splicing in H3.3^K27M^ unique/enriched interactors.

Importantly, we also identified processes that are potentially linked to the H3.3K27M CIN/GIN phenotype. For instance, the H3.3^K27M^ unique/enriched interactome showed greater heterogeneity in nucleosome composition where histone H1 variants are bound equally, yet the usage of canonical histone H2A and H2B variants is different (S2E Fig). Of note, we found an increase of macroH2A.1 (H2AY) in H3.3^K27M^, suggesting perturbed replication in H3.3^K27M^ cells [45] (S2E Fig). Moreover, we observed enrichment for replication-coupled histone chaperoning complexes (comprising ASF1B, NASP, FACT, RAN and Nucleophosmin (NPM)) in H3.3^K27M^ unique/enriched interactors [46–49] (Fig 2C and S4 Table). Furthermore, GO-term analysis identified DNA replication, including the MCM2-7 complex, as an enriched biological process specific for H3.3^K27M^ (Fig 2C, 2D and 2G) [49,50]. The latter finding was confirmed in independent pull-down experiments, where we found increased MCM2, MCM4 and MCM7 interaction with Histone H3.3K27M in mitotic cells (Fig 2H and 2I).

### H3.3^K27M^ cells are sensitive to replication stress

The enriched association of replication-specific factors with mitotic H3.3K27M may appear counterintuitive at first. However, ASF1, PCNA, other components of the MCM helicase and additional replication proteins, remain associated with chromatin in mitotic cells upon replication stress occurring during S-phase [50–53]. Hence, we hypothesized that in a H3.3K27M chromatin context, proceeding of the replication machinery upon encountering physical barriers is impaired. This hampers faithful completion of S-phase and eventually interferes with chromosome segregation [54]. To test this, we investigated if H3.3^K27M^ cells are sensitive to replication stress-inducing agents [25,26,55]. We first studied the UVC-induced DNA damage response that has been associated with H3.3 before [55], and observed a trend towards a greater UVC sensitivity for H3.3^K27M^ cells compared to H3.3^WT^ and EV (S3A Fig, left panel). In contrast, we found no differences when applying ɣ-rays (S3A Fig, right panel) [56]. We then analyzed ɣ-H2AX positivity to demonstrate DNA damage in UVC-irradiated cells using flow cytometry (S3B and S3C Fig). An S-phase specific ɣ-H2AX peak one hour following UVC induction was observed in all cell lines, which disappeared more rapidly in H3.3^K27M^ cells. This could mean that DNA damage is more rapidly resolved in H3.3^K27M^ cells, or alternatively, that DNA damage signaling ceases prematurely. Additionally, cell cycle analysis at 24 hrs after UVC showed a decrease in G2 arrested cells in H3.3^K27M^ compared to H3.3^WT^ and EV cells (S3D and S3E Fig), which could not be explained by a leaky G2 checkpoint (S3F and S3G Fig). We therefore speculate that H3.3^K27M^ cells experiencing UVC-induced DNA lesions take longer to complete replication.

### H3.3^K27M^ mutant cells exhibit increased chromosomal instability upon replication stress

**Fig 3:**
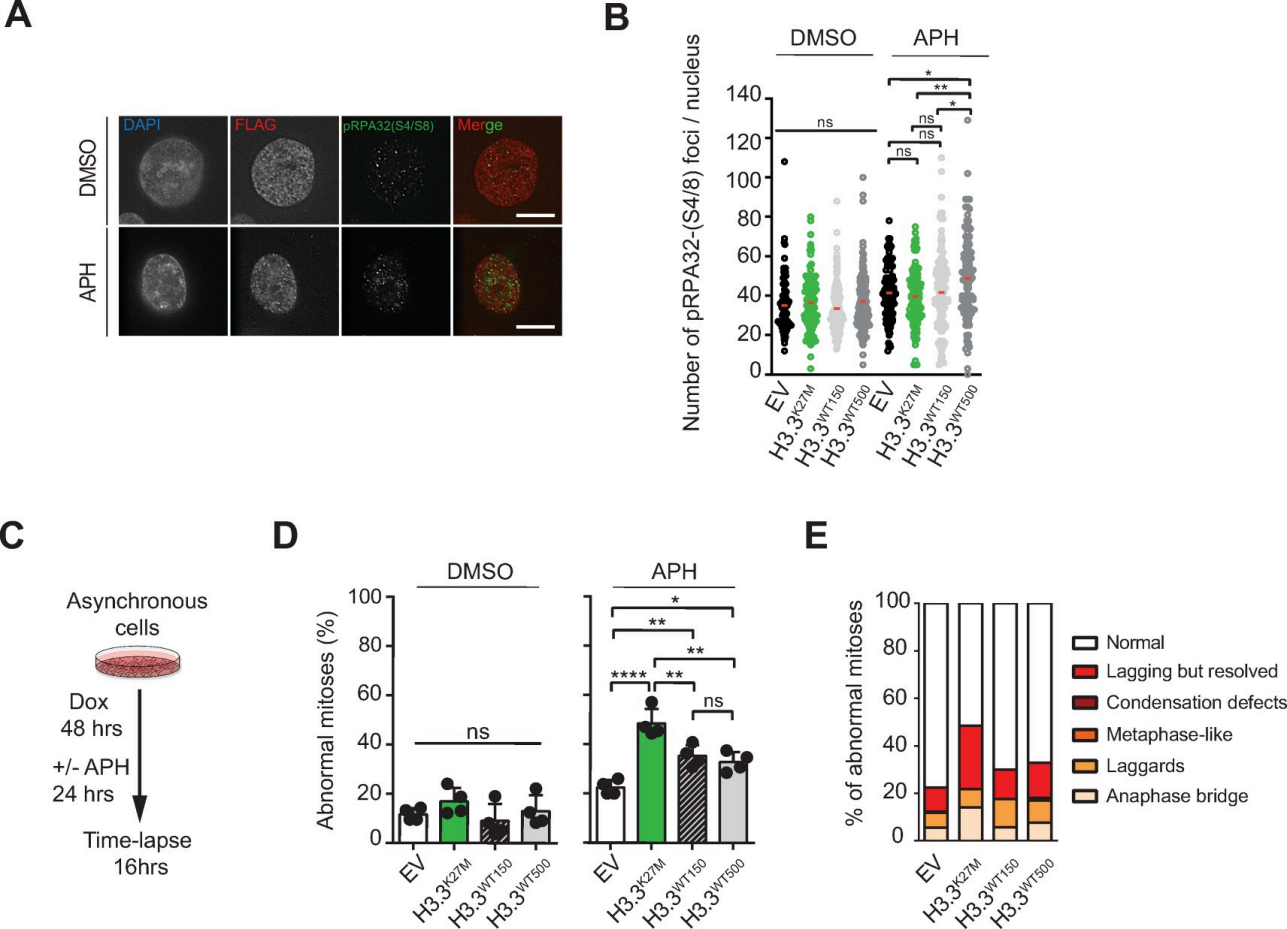
H3.3^K27M^ RPE1 cells are sensitive to replication stress. **(A)** Representative images of multiple pRPA32(S4/S8) nuclear foci in untreated and Aphidicolin (APH) treated cells. Scale bars represent 10 μm. **(B)** Quantification of the initial replication stress response by pRPA32(S4/S8) staining in DMSO or Aphidicolin (APH) treated cells. Each circle represents a single nucleus, means are represented by a red square (n = 1 experiment with > 95 nuclei per condition), *P = 0.0105 and 0.0126, **P = 0.0011 (one-way ANOVA, Tukey correction for multiple comparisons). **(C)** Experimental outline for measuring chromosomal instability by time lapse microscopy under replication stress conditions (low dose (0.2 μM) Aphidicolin (APH) treatment for 24 hrs on 48 hrs Doxycycline-treated cells). **(D)** Time lapse imaging of mitotic features following 24 hrs Aphidicolin treatment. Mitotic features were scored as normal, or abnormal. Data represent mean percentages of abnormal mitoses ± SD (n = 4 experiments), with a minimum of 20 mitoses per condition), *P = 0.0258, **P = 0.0066, 0.0056 and 0.0015, ****P<0.0001 (one-way ANOVA, Tukey correction for multiple comparisons). **(E)** Chart representing type and distribution of mitotic abnormalities after Aphidicolin (APH) treatment. For examples of scored categories, see Fig 1J.

It is unlikely that at tumor initiation, H3.3K27M mutant brain cells encounter exogenous sources of replication stress such as UVC that would provoke genomic instability. However, during development neural progenitor cells undergo phases of extremely rapid proliferation that are believed to cause significant endogenous replication stress [29–31]. To mimic this situation in the RPE1 cell model, we studied sensitivity to the chemical compound Aphidicolin (APH), which inhibits DNA polymerases leading to replication stress [57]. We first looked at phosphorylation of RPA, an early marker for replication fork stalling [58] (Fig 3A and 3B). We found that upon addition of APH, RPA phosphorylation was similar in all conditions except for H3.3^WT500^ cells that showed an increase compared to control (Fig 3B). This suggests that the H3.3^K27M^ replication stress phenotype is not caused by defects in initial replication stress signaling, but rather by altered processing of stalled forks. Unresolved replication forks cause accumulation of pre-mitotic damage and subsequent mitotic defects [59]. Therefore, to determine whether replication stress can result in an abnormal mitotic progression and CIN in H3.3^K27M^ cells, we assessed mitotic abnormalities by time lapse imaging (Fig 3C–3E). Whereas after APH treatment, an increase in abnormal mitoses was observed in all conditions, H3.3^K27M^ cells exhibited significantly more chromosome missegregation events than H3.3^WT^ or EV cells (Fig 3D). Qualitative analysis of the abnormal mitoses revealed an increase in lagging chromatin and anaphase bridges (Fig 3E). This is suggestive of persisting under-replicated DNA in mitosis, leading to DNA entanglements that ultimately cause segregation defects [60].

**Fig 4:**
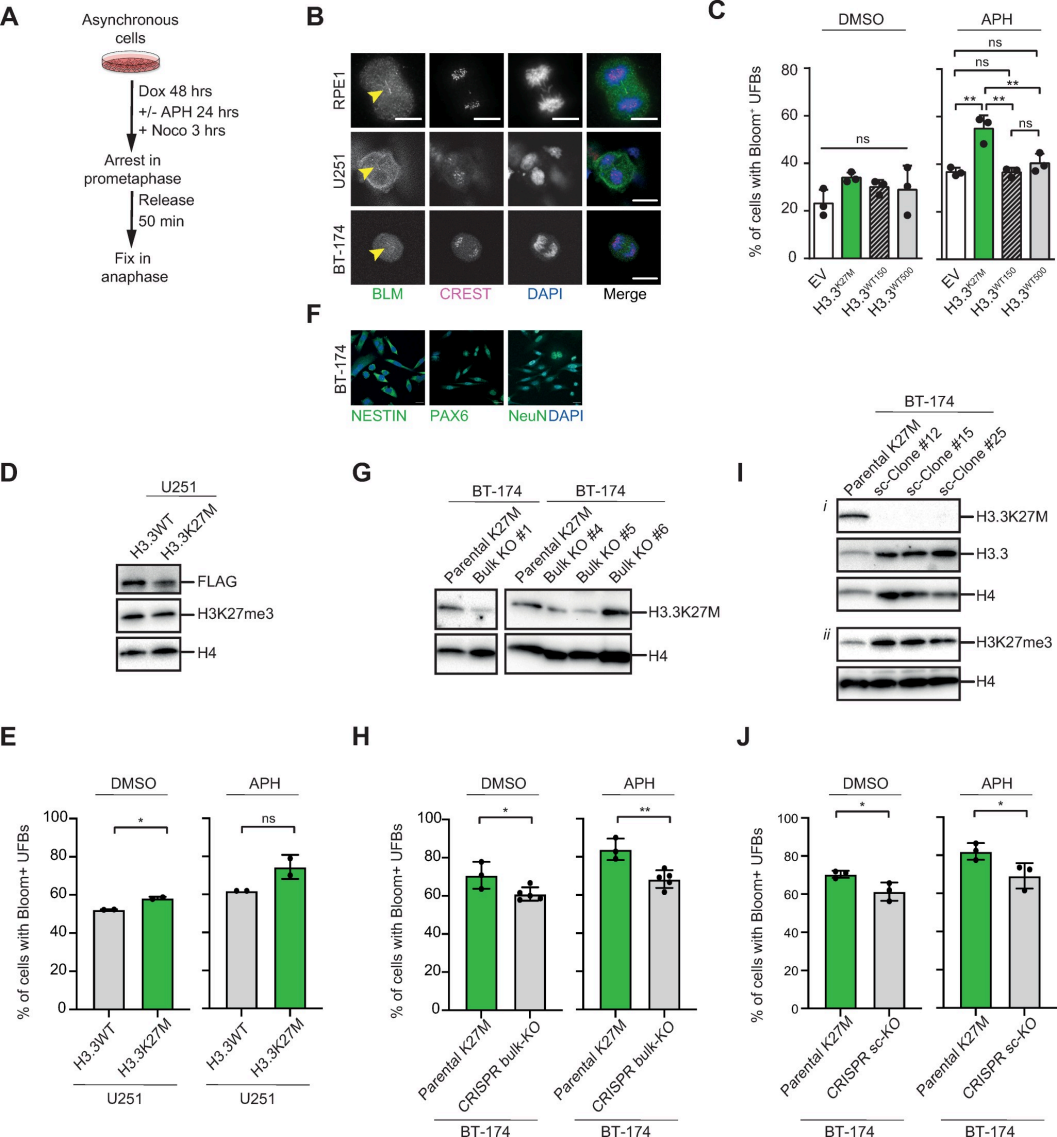
H3.3K27M affects replication stress-induced DNA ultrafine bridge (UFB) formation. **(A)** Experimental outline for determining the number of replication stress-induced DNA ultrafine bridges (UFBs) in RPE1 cells. Doxycycline induced asynchronized cells were treated with Aphidicolin (APH) for 24 hrs and subsequently blocked in mitosis with Nocodazole to enrich for mitotic cells. After release, cells were fixed in anaphase enabling visualisation of DNA UFBs. Note that for U251 and BT-174 UFB experiments, no Doxycycline pre-treatment was performed. **(B)** Confocal images showing representative examples of Bloom (BLM) coated DNA UFBs (indicated by arrowheads) in RPE1 cells (upper panels), U251 cells (middle panels) and BT-174 cells (lower panels). CREST immunolabeling reveals kinetochores, counterstaining was performed with DAPI. Scale bars represent 10 μm. **(C)** Quantification of Bloom coated DNA UFBs upon Aphidicolin (APH) and Nocodazole treatment of RPE1 cells. Data are represented as means ±SD (n = 3 experiments with +/- 30 mitoses per condition), **P = 0.0013, 0.0012 and 0.0055, (one-way ANOVA, Tukey correction for multiple comparisons). **(D)** Western blot showing FLAG and H3K27me3 expression in U251 human glioblastoma cells transfected with either H3.3WT or H3.3K27M expression constructs. H4, histone H4, loading control. **(E)** Quantification of Bloom coated DNA UFBs upon Aphidicolin (APH) and Nocodazole treatment of U251 cells. Data are represented as means ±SD (n = 2 experiments with > 20 mitoses per condition), *P = 0.0377 (paired t-test). **(F)** Confocal images showing representative stainings of neural markers in BT-174 H3.3K27M pediatric high-grade glioma cells. Counterstaining was performed with DAPI. Scale bars represent 20 μm. **(G)** Western blot showing H3.3K27M expression in BT-174 parental K27M cells or bulk BT-174 CRISPR-KO cells (Bulk KO #1, #4, #5, and #6). H4, histone H4, loading control. **(H)** Quantification of Bloom coated DNA UFBs upon Aphidicolin (APH) and Nocodazole treatment of parental BT-174 K27M cells or bulk BT-174 CRISPR-KO cells. Data are represented as means ±SD (n = 3–5 experiments with > 20 mitoses per condition), *P = 0.0357 and **P = 0.0051 (unpaired t-test). **(I)** Western blots showing (*i*) H3.3K27M and H3.3 expression, and (*ii*) H3K27me3 expression in BT-174 parental K27M cells or single-cell derived BT-174 CRISPR-KO clones (sc-Clone #12, #15, and #25). H4, histone H4, loading control. **(J)** Quantification of Bloom coated DNA UFBs upon Aphidicolin (APH) and Nocodazole treatment of parental BT-174 K27M cells or single-cell derived BT-174 CRISPR-KO clones. Data are represented as means ±SD (n = 3 experiments with > 30 mitoses per condition), *P = 0.0381 and P = 0.0497 (unpaired t-test).

Notably, a specific class of mitotic DNA structures called Ultrafine Anaphase Bridges (UFBs) has been described to form from under-replicated DNA following replicative stress [61–63]. However, unlike bulky anaphase bridges, UFBs are histone free and cannot be visualized by common DNA binding dyes, and would thus be missed in our time lapse imaging experiments. Therefore, we again treated our cells with APH, synchronized them in mitosis with Nocodazole, released them into anaphase and subsequently performed immunofluorescence staining for UFB marker Bloom (BLM) and kinetochore marker CREST (Fig 4A, 4B (upper panels), and 4C) [61,62]. This revealed a greater occurrence of BLM coated DNA fibers in H3.3^K27M^, indicating an increase in UFBs (Fig 4C). Of note, we made similar observations upon treatment with UVC (S3H Fig).

We next wished to validate these findings in a more relevant cell type. Hereto, we transfected U251 glioblastoma cells with either H3.3WT or H3.3K27M constitutive expression constructs (Fig 4D). Then, these cells were treated with APH, synchronized with Nocodazole, and analyzed for BLM coated UFBs (Fig 4B, middle panels, and 4E). We found that alike in RPE1 cells, the number of cells expressing UFBs was significantly increased in DMSO treated H3.3K27M compared to H3.3WT U251 cells, and there was a clear trend towards an increase in APH treated H3.3K27M cells (Fig 4E).

Lastly, we wanted to address if loss of the H3.3K27M mutation from tumor cells also impacts on UFB formation. Hereto, we employed primary BT-174 cells that have been derived from a pediatric diffuse midline glioma carrying a heterozygous H3.3K27M mutation in the *H3F3A* gene (Fig 4F). Using a previously established CRISPR Knockout (KO) targeting approach [64], we could show that bulk-treated BT-174 cells (further referred to as BT-174 CRISPR bulk-KO) exhibited reduced expression of H3.3K37M mutant protein, indicating successful deletion of the mutant allele in a part of the cell population (Fig 4G). When we assessed these cells for UFBs, we found that both DMSO and APH treated BT-174 CRISPR bulk-KO cells displayed a significant reduction in UFB positive nuclei (Fig 4B, lower panels, and 4H) compared to the parental cell line. In addition, we isolated single cells from the bulk CRISPR targeted populations, and obtained three single-cell derived clones (hereafter referred to as BT-174 sc-Clones) that completely lacked H3.3K27M protein expression (Fig 4I). Confirming our previous observations, also the BT-174 sc-Clones showed a reduction in cells expressing UFBs (Fig 4J).

Altogether, these data reveal a link between increased sensitivity to replication stress and mitotic aberrancies that may ultimately propagate copy number alterations in an oncohistone context.

### H3.3^K27M^ mutant cells do not induce 53BP1 nuclear bodies in response to UFBs

**Fig 5:**
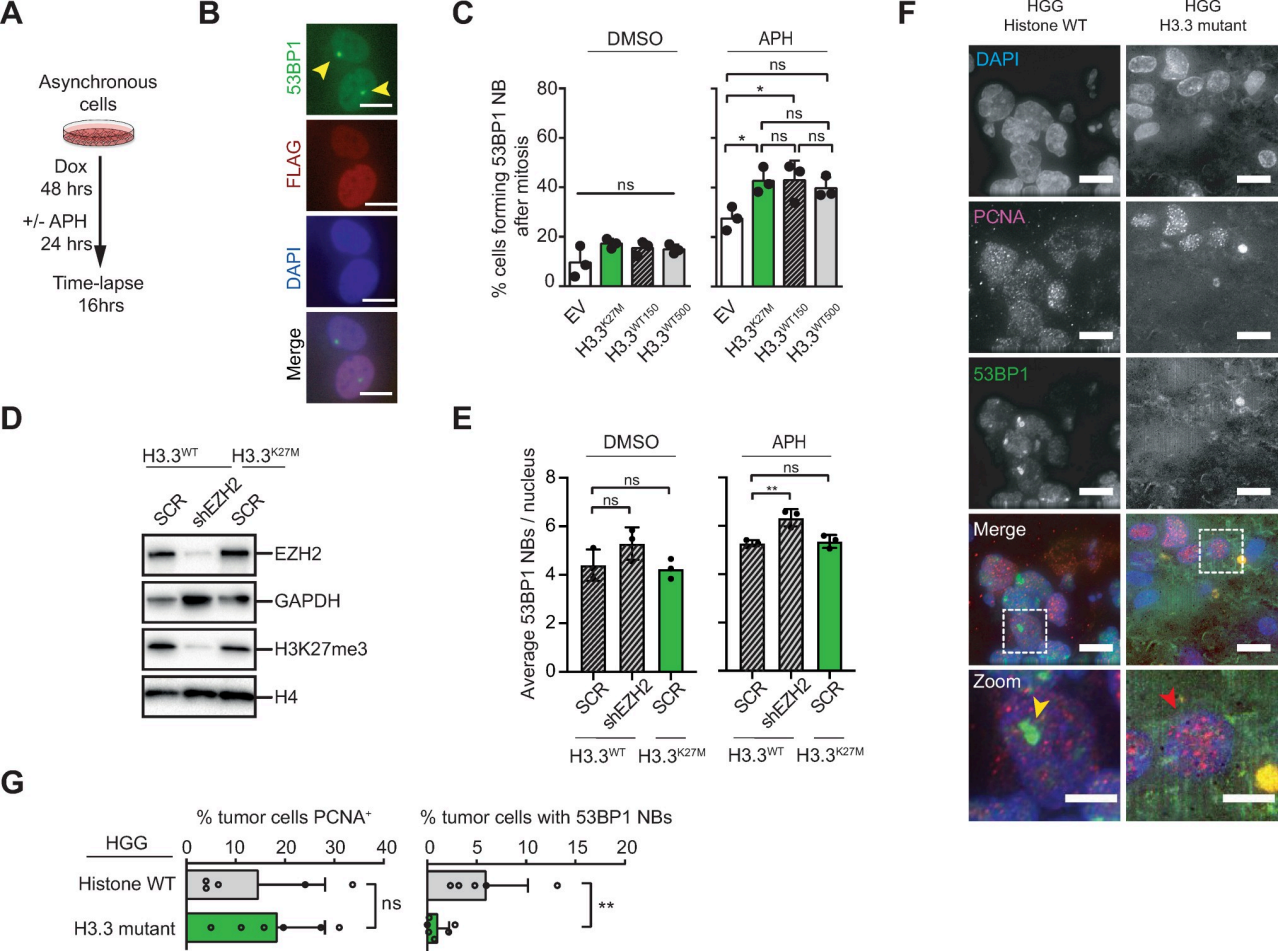
Altered 53BP1 response in H3.3^K27M^ cells and high-grade glioma. **(A)** Experimental outline for measuring 53BP1 nuclear body formation by time lapse microscopy in replication stress conditions (Low dose Aphidicolin (0.2 μM) treatment for 24 hrs following 48 hrs of Doxycycline induction). **(B)** Immunofluorescent images depicting typical mirrored 53BP1 nuclear bodies in two daughter cells commonly seen after rupture of an unresolved DNA UFB. Scale bars represents 10 μm. (**C)** Quantification of 53BP1 nuclear bodies (NB) following 24 hrs Aphidicolin treatment by time lapse microscopy. Chart representing the percentage of cells with 53BP1 nuclear body formation after mitosis. Data are represented as means ±SD (n = 3 experiments with > 20 mitoses per condition), *P = 0.0463 and 0.0430 (one-way ANOVA, Tukey correction for multiple comparisons). **(D)** Western blot showing EZH2, GAPDH and H3K27me3 expression in H3.3^WT^ or H3.3^K27M^ RPE1 cells treated with a scrambled shRNA (SCR), or an shRNA against EZH2 (shEZH2). H4, histone H4, loading control. **(E)** Quantification of 53BP1 nuclear bodies (NB) following 24 hrs Aphidicolin treatment of H3.3^WT^ or H3.3^K27M^ RPE1 cells treated with a scrambled shRNA (SCR), or an shRNA against EZH2 (shEZH2). Chart representing the average number of 53BP1 nuclear bodies per nucleus. Data are represented as means ±SD (n = 3 experiments with > 70 nuclei per condition), **P = 0.0055 (one-way ANOVA, Dunnett correction for multiple comparisons). **(F)** Representative images of two untreated pediatric HGG biopsy samples stained for proliferation marker PCNA (red arrows) and 53BP1 (yellow arrows) to reveal the nuclear body load. Dashed squares indicate the zoomed area. Scale bar represents 10 μm (5 μm on zooms). **(G)** Quantification of the proliferation rate (*i*.*e*., percentage of PCNA positive cells) and 53BP1 nuclear body (NB) load (percentage 53BP1 positive cells) per HGG tumor biopsy. Each circle represents the number for one tumor (HGG Histone^WT^, n = 5; HGG Histone^H3.3 Mutant^, n = 6). Data are represented as means per histone group ±SD (with > 200 cells analyzed per tumor). **P = 0.0087 (Mann Whitney non-parametric t-test).

Unresolved UFBs tend to break during mitotic exit [65]. These breaks lead to symmetrically inherited DNA damage in daughter cells, which co-occurs with large nuclear bodies containing the p53 Binding Protein-1 (53BP1) and other proteins [66,67]. To study 53BP1 nuclear body (53BP1 NB) formation following APH treatment, we generated a new set of inducible histone cell lines co-expressing a 53BP1-EGFP fusion protein and performed time lapse imaging (Fig 5A and 5B). In line with the increase in UFBs (Fig 4), we observed an increase in 53BP1 NBs following APH treatment in all conditions (Fig 5C). Yet surprisingly, H3.3^K27M^ cells did not show a higher increase in 53BP1 NB formation compared to H3.3^WT^ or EV cells (Fig 5C). This either suggests that the increased replication stress is timely resolved, or alternatively, that the 53BP1 response following UFB formation is inadequate in H3.3^K27M^ cells, as we expected more 53BP1 NBs in H3.3^K27M^ cells due to the higher incidence of UFBs. Since we did not observe an impaired 53BP1 NB response in RPE1 cells containing an shRNA construct targeting EZH2 (Fig 5D and 5E), it is unlikely that the inadequate 53BP1 response found in H3.3^K27M^ cells is caused by changes in EZH2 activity or subsequential alterations in H3K27me3 expression [68]. To further address this in an oncogenic setting, we looked at the expression of 53BP1 NBs in a panel of pediatric HGG biopsies taken at diagnosis, which were either histone wild type (WT-HGG, n = 5), or H3.3 mutant (H3.3-HGG, n = 6) (Fig 5F and 5G). We found that whereas H3.3 mutant gliomas were highly proliferative as revealed by PCNA staining (Fig 5G, left panel), they exhibited low 53BP1 NB counts in comparison to histone wild-type glioma biopsies (Fig 5G, right panel). This corroborates an inadequate 53BP1 NB response in the presence of the H3.3K27M oncohistone.

## Discussion

Whereas the changes in epigenetic patterning in histone mutant gliomas have been extensively investigated [1–6,9], the exact mechanisms driving oncogenesis remain elusive to date. Rather than being solely dependent on transcriptional changes, we hypothesize that pediatric high-grade glioma results from an interplay of multiple pro-tumorigenic processes that are related to the different functions of the multifaceted (onco)histone H3.3. These include H3.3 as an epigenome/transcriptome regulator, but also as an important actor in different DNA damage repair mechanisms and in maintenance of genome integrity, which can explain the tendency for H3.3 mutant gliomas to be highly aneuploid [4,16,24,69–71]. The latter idea was explored in this study, in which we discovered that histone mutant H3.3K27M cells are more susceptible to acquire genome instability resulting from an intrinsic sensitivity to stress during DNA replication.

While studying the mechanism underlying the strong genomic instability found in H3.3K27M HGG, we made a number of observations that point at H3.3K27M deregulating several processes that may be related. For instance, the more rapid resolution of the ɣ-H2AX signal following UVC exposure could indicate that DNA damage signaling ceases prematurely in the presence of the H3.3K27M mutant histone. This could allow damaged cells to pass the G2 cell cycle checkpoint and progress into mitosis. Interestingly, in our mitotic pulldown experiments, we uncovered an enrichment for DNA replication components, comprising PCNA, Mini-Chromosome-Maintenance proteins (MCMs) and replicative histone chaperones, in H3.3^K27M^ cells. This was unexpected, as although MCM proteins have been identified as direct histone H3 interactors previously, these factors are typically unloaded prior to mitosis [49,50,72,73]. Under physiological conditions, the MCM complex assembles in G1 as a pre-replisome complex on DNA in a process called origin licensing [74]. In late G1 and S-phase, interaction with the replicative helicase converts the structure into the replicative-complex that initiates replication, or origin firing [75]. Of note, MCM complexes are widely deposited throughout the genome, but most remain dormant and only become activated during replication stress to ensure complete genome duplication [76–78]. When this process fails, PCNA and MCMs remain stably locked with un-replicated chromatin throughout the cell cycle until the next S-phase, and this might lead to MCM binding to mitotic DNA [50,51,79,80]. Thus, the increased binding of MCM proteins to H3.3K27M may result from stalled replication forks that were not resolved prior to mitosis. The reason for this remains speculative, but it is possible that replication stress-induced damage remained undetected until mitosis due to improper DNA damage signaling. Alternative explanations are H3.3K27M-induced changes in the epigenetic landscape impairing origin licensing or firing; or altering the distribution of H3.3 proteins to alternative genomic localizations, the latter potentially leading to interactions with novel binding partners. Yet it is also possible that the Lysine 27 substitution directly interferes with MCM binding [49,50,72,73,81,82].

Additional evidence for unresolved DNA replication problems in mitotic H3.3K27M cells is provided by the increased numbers of DNA UFBs. Normally, mitotic DNA synthesis (MiDAS) of under-replicated DNA provides the lattermost opportunity to complete DNA replication and therefore tackle extensive genome instability [51]. Hence, it is possible that MiDAS is also impaired in H3.3K27M cells, especially since H3.3K27M inhibits the EZH2 histone methyltransferase that places the H3K27me3 mark that serves as docking platform for key MiDAS factor MUS81 [10,15,51,83–87]. Furthermore, it is quite remarkable that relatively small changes in H3.3K27M expression can have such profound effects on UFB formation. In our RPE1 cell model, the fraction of mutant H3.3 was around thirty percent of endogenous H3.3. This is largely in line with what has been reported for tumors *in vivo*, in which mutant H3.3 constitutes up to twenty percent of all H3.3—yet with a dramatic effect on outcome [10]. Importantly, when we deleted the mutant *H3F3A* allele from primary H3.3K27M HGG cells, leaving the three wild type alleles intact (*i*.*e*, one remaining copy of *H3F3A* and two copies of *H3F3B*), the number of UFBs decreased significantly. This suggests that the H3.3K27M oncohistone, despite relatively low expression, remains an important driver of genomic instability even in a malignant background.

Finally, we found that the increase in UFBs did not trigger an increased recruitment of 53BP1 nuclear bodies in H3.3^K27M^ cells, suggesting that not all UFB breaks are buffered by this salvage mechanism. This observation was further accentuated by the H3.3 mutant tumors, which exhibited relatively low numbers of 53BP1 nuclear bodies compared to histone wild type tumors. Indeed, H3.3 gliomas tend to co-mutate DNA repair enzymes that also have a role in 53BP1 nuclear body formation, which could suggest that bypass of the 53BP1 response is favorable for H3.3K27M (tumor) cells as disruption of such a repair mechanism might prevent resolving the DNA damage resulting from UFBs and thus enhance genome plasticity and heterogeneity of the tumor, and ultimately help the cancer cells to thrive [4].

In conclusion, efficient targeting of H3.3 mutant pediatric high-grade glioma remains highly challenging and it is likely that the multi-faceted functions of (mutant) H3.3 play an important part in this. The findings presented in this study highlight a few of these functions and provide a possible explanation for the GIN/CIN phenotype observed in H3.3K27M high-grade glioma. They also suggest that these tumors might be more susceptible to compounds that exacerbate replication stress, however this should be investigated in follow up studies.

## Materials and methods

### Ethics statement

All pediatric brain tumor tissue was obtained following surgical resection at diagnosis.

Pediatric H3.3K27M HGG samples subjected to single-cell WGS were obtained from the University Medical Center Groningen, the Netherlands, and the University Medical Center Hamburg-Eppendorf, Germany. Histone wild type and mutant HGG samples subjected to immunostainings were obtained from the University Medical Center Hamburg-Eppendorf, Germany.

For brain tumor tissue obtained from the University Medical Center Groningen, informed consent was given by parents/guardians of the patient for the collection of brain tumor samples. The local Medical Ethics Committee (METc/Medisch Etische Toetsingscommissie) of the University of Groningen was informed and the study was not liable to the WMO (Wet Medisch-wetenschappelijk Onderzoek met mensen/ Medical Research involving human subjects act).

Brain tumor tissue obtained from the University Medical Center Hamburg, Eppendorf, was collected for diagnostic purposes and afterwards used in an irreversibly anonymized manner. This approach was fully covered by the local rules stated in the Hamburg Hospital law (Hamburgisches Krankenhausgesetz, HmbKHG, §12) and does not require specific patient consent or approval from the local ethics committee.

Primary H3.3K27M brain tumor cells BT-174, derived from a H3.3K27M pediatric diffuse midline glioma of the brain stem, were obtained from the Princess Máxima Center for Pediatric Oncology. Experiments with this human material were approved by the Medical Ethics Committee (METc) of the Erasmus Medical Center (Rotterdam, the Netherlands) and Princess Máxima Center for Pediatric Oncology (Utrecht, the Netherlands). Written informed consent was obtained from the patient and/or parents/guardians of all participants (MEC-2016-739).

### DNA copy number analysis

Distribution of DNA copy number alterations (CNAs) of pediatric high-grade gliomas was determined using a publicly available dataset of pediatric high-grade gliomas consisting of 4 subtypes: H3.3G34R/V mutant (n = 63), H3.3K27M mutant (n = 245), H3.1/H3.2K27M mutant (n = 50), and H3 wild type (n = 387) [4]. Autosomal copy number aberrations per tumor were summed and CNA distribution was compared between tumor subtypes (Mann Whitney). Copy number alterations had already been determined previously by Mackay et al [4].

### Single-cell whole genome sequencing and data analysis

A single cell suspension was freshly prepared by trituration of the resected tumor tissue and subsequent digestion with Accutase (Gibco). The cell suspension was cryopreserved in 10% DMSO. For single nuclei sorting, cells were resuspended in staining buffer (1M tris-HCl pH7.4, 5M NaCl, 1M CaCl_2_, 1M MgCl_2_, 7.5% BSA, 10% NP-40, ultra-pure water, 10 mg/ml Hoechst 33358, 2 mg/ml propidium iodide), and kept on ice in the dark to facilitate lysis. G1 single nuclei, as assessed by PI and Hoechst staining, were sorted into 96 wells plates on a MoFlo-Astrios flow cytometer (Beckman Coulter). Nuclei were lysed and DNA was barcoded, followed by automated library preparation (Agilent Bravo robot) as described previously [88]. Single cell libraries were pooled and sequencing was performed using a NextSeq 500 machine (Illumina; up to 85 [HGG1] or 77 cycles [HGG2 and HGG3]; single end). The generated data were subsequently demultiplexed using sample specific barcodes and changed into fastq files using bcl2fastq (Illumina; version 1.8.4). Reads were afterwards aligned to the human reference genome (GRCh38/hg38) using Bowtie2 (version 2.2.4) [89]. Duplicate reads were marked with BamUtil (version 1.0.3) [90].

The aligned read data (bam files) were analyzed with the copy number calling algorithm AneuFinder [91]. Following GC correction and blacklisting of artefact-prone regions (extreme low or high coverage in control samples), libraries were analyzed using the dnacopy and edivisive copy number calling algorithms with variable width bins (average binsize = 1 Mb; step size = 500 kb). The three samples were analyzed with an euploid reference [88].

Results were afterwards curated by requiring a minimum concordance of 90% between the results of the two algorithms. Libraries with on average less than 5 reads per bin and per chromosome copy (~ 30,000 reads for a diploid genome) were discarded. Anonymized reads were deposited at the European Nucleotide Archive/ENA (https://www.ebi.ac.uk/ena/browser/home), accession number PRJEB46622. The aneuploidy, structural and heterogeneity scores were calculated as follows.

The aneuploidy score of each bin was calculated as the absolute difference between the observed copy number and the expected copy number when euploid. The score for each library was calculated as the weighted average of all the bins (size of the bin as weight) and the sample scores were calculated as the average of the scores of all libraries.

The heterogeneity score of each bin was calculated as the proportion of pairwise comparisons (cell 1 vs. cell 2, cell 1 vs cell 3, etc.) that showed a difference in copy number (*e*.*g*. cell 1: 2-somy and cell 2: 3-somy). The heterogeneity score of each sample was calculated as the weighted average of all the bin scores (size of the bin as weight).

The structural score of each library was calculated as the number of detected breakpoints (number of copy number transitions) divided by the total genome length in Mb (average number of breakpoints per Mb). The structural score of each sample was calculated as the average structural score of all libraries.

### Cloning of (retro/lentiviral) expression constructs

pcDNA4/TO-FLAG-H3.3 was a gift from Bing Zhu (Addgene plasmid # 47980; http://n2t.net/addgene:47980; RRID:Addgene_47980) [92]. pCDNA4/TO-FLAG-H3F3A-K27M mutant was generated using Q5 Site-Directed Mutagenesis Kit (New England Biolabs Inc.) according to the manufacturer’s recommendations. Mutagenesis primers used were designed using NEBaseChanger: Forward: GCCGCTCGCAtGAGTGCGCCC; Reverse: TTTTGTAGCCAGTTGCTTCCTGGG). Vectors were sequenced to validate the presence of the mutation *A(A>T)G (nucleotide number 296)* (CMV^Fwd^: CGCAAATGGGCGGTAGGCGTG, BGH^Rev^: TAGAAGGCACAGTCGAGG). Both WT and mutant cloning vectors were digested with BamHI and EcoRI to obtain the FLAG-H3F3A-WT and FLAG-H3F3A-K27M containing inserts that were subsequently cloned into pLVX-Tight-puromycin or blasticidin (Clontech).

For generating pCDNA-FLAG-hH3F3A(WT/K27M) allowing constitutive expression, inserts from pCDNA4/TO-FLAG-H3F3A(WT/mutant) were cloned into BamHI and EcoRI digested pCDNA3 (Invitrogen). Clones were validated by Sanger sequencing (CMV Fw: ATGGGCGGTAGGCGTGTACGGTGGGAG; SV40 Rv: ACCCTAACTGACACACATTCC).

For generating the EZH2 knockdown construct, the following sequence for EZH2 shRNA was taken from MISSION shRNA: TRCN0000018365 (EZH2-shRNA) and ordered as oligonucleotides (IDT). Cloning of the shRNA was performed as described before using pLKO.1 –TRC cloning vector BamHI and EcoRI sites [93,94]. All constructs were validated using Sanger sequencing (hU6 Fw: GAGGGCCTATTTCCCATGATT). pLKO.1 - TRC cloning vector was a gift from David Root (Addgene plasmid # 10878; http://n2t.net/addgene:10878; RRID:Addgene_10878) [95]. pLKO.1 construct containing a scrambled shRNA was a gift from David Sabatini (Addgene plasmid # 1864; http://n2t.net/addgene:1864; RRID:Addgene_1864) [96].

### Cell culture conditions and retro/lentiviral transductions

All human RPE1-hTERT immortalized cell lines (hereafter referred to as RPE1) (ATCC) were cultured in DMEM (Gibco) + 10% FBS (Gibco) and 1% penicillin-streptomycin (Gibco), and were never passaged above 30 passages. Doxycycline inducible RPE1 cell lines were generated at the same time by retroviral transduction of pRetrox-rtTA with subsequent Geneticin selection. The pLVX-Tet-On system was used and cells were transduced with either pLVX-tight-puro (hereafter referred to as empty vector/EV), pLVX-tight-puro-FLAG-H3F3A-WT (H3.3^WT^) or pLVX-tight-puro-FLAG-H3F3A-K27M (H3.3^K27M^) lentivirus and selected with Puromycin. For visualization of 53BP1 nuclear body formation, cell lines were transduced with the pLNCX-53BP1-EGFP construct kindly provided by Dr. Marcel van Vugt. The transduced population was enriched by sorting for GFP positive cells. For EZH2 knockdown experiments, RPE1 cells containing pRetrox-rtTA were transduced with pLVX-tight-blasticidin empty vector (EV), pLVX-tight-blasticidin FLAG-hH3F3A-WT (H3.3^WT^), or pLVX-tight-FLAG-hH3F3A-K27M (H3.3^K27M^) lentivirus and selected with Blasticidin (Sigma). Cells were subsequently transduced with pLKO-Puro-shRNA-EZH2 or scrambled. All cell lines were tested free of mycoplasma.

U251 glioblastoma cells (Sigma) were cultured in DMEM (Gibco), 10% FBS (Gibco) and 1% penicillin-streptomycin (Gibco).

Early passage BT-174 cells were cultured in TSM medium (DMEM-F12 (Gibco) 1: 1 Neurobasal-A medium (Gibco), 10 mM HEPES (Gibco), 2 mM Glutamax (Thermo), 1 mM Sodium Pyruvate (Gibco), 0.1 mM Non-essential amino acids (Gibco), 2.5% FBS (Gibco), 1% Antibiotic-Antimycotic solution (Gibco), 0,1% β-mercaptoethanol (Gibco), 1% N2 supplement (Gibco), 2% B27 supplement without vitamin A (Gibco), 20 ng/mL hEGF, 20 ng/mL hFGF-2, 10 ng/mL PDGFAA, and 10 ng/mL PDGFBB (Peprotech)).

### CRISPR/Cas9-mediated knockout of H3.3K27M

CRISPR/Cas9 genome editing on BT-174 cells was carried out with the gRNA/Ribonucleoprotein (RNP) delivery strategy, as previously described [97]. Briefly, gRNA duplex was prepared with Alt-R crRNA XT (IDT) and Alt-tracrRNA-ATTO550 (IDT). Oligonucleotides were reconstituted to 200 μM with Nuclease-free Duplex buffer (IDT), mixed at equimolar concentrations (80 μM), annealed at 95°C for 5 min and cooled down to RT. The RNP complex was prepared by mixing 125 pmol of gRNA duplex with 62,5 pmol of Alt-R S. p. HiFi Cas9 nuclease v3 (IDT) and incubated for 20 min at RT. The specific gRNA sequence used is described elsewhere [64]. BT-174 cells were nucleofected with RNP complex using the P3 Primary Cell 4D-nucleofector X kit, according to the manufacturer’s instructions and technical support (Lonza). Shortly, one million cells were resuspended in nucleofection solution, mixed with RNP complex and transferred to a 100 μL single cuvette. Electroporation was carried out on a 4D nucleofector X unit (program CA-189). Immediately following nucleofection, cells were resuspended in TSM medium and plated. After 48 hrs, ATTO550 positive cells were sorted as single cells into 96 well plates for generating single cell clones, or as bulk cell populations into tubes using the Sony SH800S cell sorter (130 μm nozzle). After expansion, single-sorted cell clones were screened for loss of the *H3F3AK27M* mutant allele using Sanger sequencing. Insertions or deletions (indels) in the *hH3F3A* gene were verified with the Synthego Inference of CRISPR Edits (ICE) analysis tool version 2.0 [98]. Loss of H3.3K27M protein expression was confirmed using Western blot analysis.

### Immunoprecipitation, LC/MS-MS analysis, label-free quantification and STRING analysis

H3.3^WT^, H3.3^K27M^ or EV cell lines were used for FLAG pull-down of H3.3 interacting proteins from total protein extracts of synchronized mitotic fractions. Hereto, cells were incubated with Doxycycline (500 ng/ml, Sigma D9891) for 48 hrs. Subsequently, cells were treated with Nocodazole (100 ng/ml, Sigma M1404, 8 hrs) after which a mitotic shake off was performed. Mitotic cells were kept at 4°C unless stated otherwise. Mitotic cell pellets were carefully washed with ice-cold PBS and resuspended in immunoprecipitation buffer containing 20 mM Tris at pH7.4, 100 mM NaCl, 5 mM MgCl_2_, 0.1% Triton X-100, 10% glycerol, 1 mM DTT, Complete protease inhibitors (EDTA-free, Roche 11873580001), phosphatase inhibitors (PhosSTOP, Roche 4906845001) and given 30 strokes with a douncer (Tissue grinder set 2ml, Sigma D8938). DNA was digested with 1U/μl OmniCleave Endonuclease (Epicentre OC7850K) for 30 minutes. The insoluble fraction was removed by centrifugation at 15,000 rpm for 10 min. Supernatant was subjected to immunoprecipitation with α-FLAG-M2 magnetic beads (Sigma, M8823) for 2 hrs with gentle rotation. Beads were washed three times with TBS. For subsequent mass-spectrometry analysis, precipitated proteins were first eluted by two rounds of competition with 3X FLAG Peptide (50 μl, Sigma F4799). Then, beads were boiled in loading buffer without DTT to elute remaining proteins. For Western blot, precipitated proteins were eluted in sample buffer (50 mM DTT).

Eluates were submitted to an in-gel tryptic digestion and proteins were separated by LC/MS-MS (Ultimate 3000 nanoHPLC Liquid Chromatography instrument (Dionex) coupled to an LTQ-Orbitrap XL (Thermo Fisher Scientific) mass spectrometer instrument). Four technical replicates were performed per condition. Protein identification and data processing was performed using PEAKS 8.5 open-source software. To quantify the protein interactome, a label free quantification method using the spectral counts of each identified interactor was performed. Nucleosomal content and pull-down efficiency was assessed based on histone H4 spectral counts (H4^spec/c^) and technical replicates with H4^spec/c^ above background (e.g. average EV [H4^spec/c^]) were kept for further analysis. Spectral counts in the EV cell line were considered as background and therefore subtracted from the spectral counts in H3.3^WT^ and H3.3^K27M^ samples. Output data were corrected for nucleosomal content by normalization to the H4^spec/c^ of the H3.3^WT^ sample.

Average spectral counts per precipitated protein were compared between H3.3^WT^ and H3.3^K27M^ samples. Precipitated proteins were classified either as *unique binding partners*, *common/not enriched binding partners* (when the difference between H3.3^WT^-[Partner^spec/c^] and H3.3^K27M^-[Partner^spec/c^] was ≤2 spectral counts), or *common/enriched binding partners* (when the difference between H3.3^WT^-[Partner^spec/c^] and H3.3^K27M^-[Partner^spec/c^] was >2 spectral counts). For identification of enriched networks, protein lists were submitted to STRING database (www.string.org). Pathway and reactome analysis were performed with the highest degree of confidence (strength of data support = 0.9) and disconnected nodes were hidden. For clustering, Markov Clustering (MCL) method was used with an inflation degree of 1.5. The illustrated network maps were simplified by manual curation for clarity.

### Cell culture treatments

For time lapse imaging of mitotic RPE1 cells, H3.3^WT^, H3.3^K27M^ or EV cells were seeded onto Lab-Tek II chambered coverglass (Thermo Fisher). Expression of FLAG-H3.3^WT^ was induced with 150 and 500 ng/ml Doxycycline or with 500 ng/ml Doxycycline for FLAG-H3.3^K27M^ for 24–48 hrs. For replication stress induction, cells were further treated with 24 hrs Aphidicolin (0.2 μM, Sigma). Two hrs prior to imaging, cells were refreshed with media containing 150 or 500 ng/ml Doxycycline and 20 nM fluorescent DNA binding dye (SirDNA, Spirochrome TebuBio) [99]. Cells were imaged for 16 hrs on a DeltaVision Elite imaging station (Applied Precision, GE Healthcare). Movies were deconvolved using SoftWork suite and analyzed with ImageJ. The assessment of abnormal mitoses or 53BP1 nuclear body formation was done manually by two independent observers using Fiji/ImageJ software.

Replication stress was visualized and quantified by staining for phosphorylated RPA32(S4/S8) positive nuclear foci. RPE1 cells were treated with Doxycycline for 48 hrs before addition of Aphidicolin or DMSO (0.2 μM, Sigma). After 24 hrs Aphidicolin (or DMSO) treatment, cells were fixed with 4% paraformaldehyde (Sigma) and stained. pRPA foci were visualized on a DeltaVision Elite imaging station (Applied Precision, GE Healthcare), a Leica SP8 or a LeicaSP8X DLS confocal microscope. Cells were identified by DAPI and FLAG staining. Pictures were deconvolved using SoftWork suite and analyzed with ImageJ. pRPA foci were counted in an automated fashion using CellProfiler software (v3.1.9).

For visualization of DNA ultrafine bridges coated with Bloom helicase protein in RPE1 cells, EV and H3.3^K27M^ were first treated with 500 ng/ml Doxycycline; and H3.3^WT^ with 150 ng/ml and 500 ng/ml Doxycycline for 48 hrs, then Aphidicolin (0.2 μM, Sigma) or DMSO was added for 24 hrs. Treated cells were synchronized in prometaphase with Nocodazole (100 ng/ml, Sigma) for 3 hrs and subsequently released, fixed in anaphase and stained.

For visualization of DNA ultrafine bridges in RPE1 cells after UVC radiation, 48 hrs Doxycycline induced cells were washed thoroughly with PBS and irradiated with UVC (4 J/m^2^, UVC500 Crosslinker, Amersham Biosciences Corp.) or they were mock treated (control). Media was refreshed and cells were synchronized in prometaphase with Nocodazole (100 ng/ml, Sigma) for 3–4 hrs and subsequently released, fixed in anaphase and stained.

For visualization of DNA ultrafine bridges in U251 cells, cells were serum-starved overnight and transfected with pcDNA3-FLAG-H3.3(WT) or pcDNA3-FLAG-H3.3(K27M) at the ratio of FUGENE 3:1 DNA (Promega). 24 hrs after transfection, cells were treated for 24 hrs with 0.2 μM Aphidicolin or DMSO. They were synchronized in prometaphase with Nocodazole (100 ng/ml, Sigma) for 3 hrs and subsequently released, fixed in anaphase and stained.

For visualization of DNA ultrafine bridges in BT-174 cells, cells were treated for 24 hrs with 0.2 μM Aphidicolin or DMSO. They were synchronized in prometaphase with Nocodazole for 3 hrs and subsequently released, fixed in anaphase and stained. Bloom positive ultrafine bridges were visualized on a Leica SP8 or SP8X DLS confocal microscope and analyzed and scored manually with ImageJ.

For visualizing 53BP1 nuclear bodies in RPE1 cells, cells were seeded on glass coverslips and treated with 500 ng/ml Doxycycline (H3.3^K27M^) or 100 ng/ml (H3.3^WT^) Doxycycline for 48 hrs. Next, they were treated with Aphidicolin (0.2 μM, Sigma) or DMSO for 24 hrs, followed by fixation. 53BP1 nuclear bodies were visualized on a Leica SP8 confocal microscope and images were processed with ImageJ. 53BP1 bodies were counted in an automated fashion using CellProfiler software (v3.1.9).

To perform ɣ-ray sensitivity assays, 48 hrs Doxycycline induced RPE1 cells were seeded into 96 wells plates and irradiated with ɣ-rays (0, 0.5 and 2 Gy, IBL 637 Cesium-137γ-ray machine) the following day. Cells were allowed to proliferate for an additional 7 days.

For UVC sensitivity assays, 48 hrs Doxycycline induced cells were washed thoroughly with PBS and irradiated with UVC (0, 1 and 7 J/m^2^, UVC500 Crosslinker, Amersham Biosciences Corp.). Cells were then replated into 96 wells plates and grown for 7 days. Doxycycline was refreshed every 48 hrs. At day 7, cells were fixed with 8% paraformaldehyde, washed with PBS and stained with crystal violet. Crystal violet was dissolved in 1% SDS solution and absorbance (595 nm) was measured.

### Immunofluorescence labeling of cells

For immunofluorescence stainings, cells were seeded onto 13 mm glass coverslips prior to treatments. They were fixed with 4% paraformaldehyde/PBS, permeabilized with 0.25% Triton X-100 and blocked with 5% BSA, 0.05% Tween20 (Sigma) in PBS. Cells were subsequently incubated overnight at 4°C using the following primary antibodies diluted in 1% BSA 0.05% Tween-20 in PBS: Mouse anti-FLAG (1/1000, Sigma F1804), Rabbit anti-Bloom (1/200, Abcam Ab2179), Human anti-CREST (1/1000, Fitzgerald, 90C-CS1058), Rabbit anti-53BP1 (1/200, Cell Signaling 4937), Rabbit anti-phosphoRPA32(S4/S8) (1/500, Bethyl A300-245A), Rabbit anti-Pax6 (1/500, Biolegend, 901301), Mouse anti-Nestin (1/1000, BD Science, 611658, and Mouse anti-NeuN (1/1000, Abcam, 104224). Secondary antibodies were goat anti-mouse Alexa-488, goat anti-rabbit Alexa-488, goat anti-mouse Alexa-568 and goat anti-human Alexa-633 (1/500, Invitrogen). Counterstaining was performed with DAPI. All coverslips were mounted using Vectashield (Vector laboratories) or Prolong Diamond Antifade Mountant (Invitrogen). Images were taken on a Leica SP8 or SP8X DLS confocal microscope.

### Immunofluorescence labeling of pediatric high-grade glioma samples

For immunofluorescence stainings, 2 μm serial paraffin sections were deparaffinized with xylene, rehydrated with ethanol 100%, 96% and 70% and water. Antigen retrieval was performed using citrate buffer. Sections were blocked with 5% normal goat serum and 0.1% Triton X-100 in PBS and incubated overnight with anti-53BP1 antibody (1/100, Cell Signaling 4937) and anti-PCNA antibody (1/2000, Abcam Ab29). Secondary antibodies were goat anti-mouse Alexa-568 and anti-rabbit Alexa-488 (Invitrogen). Sections were counterstained with DAPI and mounted with Vectashield (Vector laboratories). Sections were imaged on a DeltaVision Elite imaging station (Applied Precision, GE Healthcare). Images were deconvolved using SoftWork suite, and blinded and randomized for analysis with ImageJ.

### Western blot analysis

Total protein extracts and pulled-down samples were boiled in sample buffer and loaded onto Mini-PROTEAN TGX precast gels (Biorad). Proteins were transferred to a PVDF membrane (Trans-Blot Turbo Transfer System, Biorad), and probed for the following antibodies: Mouse anti-FLAG (1/1000, Sigma F1804), Mouse anti-total H3 (1/1000, Cell Signaling 3638), Rabbit anti-Histone H3.3 (1/1000, Clone RM190 Rev mab biosciences 31-1058-00), Rabbit anti-H3K27me^3^ (1/1000, Cell signaling 9733), Mouse anti-H4 (1/1000, Active motif 61521), Rabbit anti-H3.3K27M (1/1000, Merck ABE419), Rabbit anti-H3K27me^3^ (1/1000, Merck 07–449), Rabbit anti-EZH2 (1/1000, Cell signaling 5246), Mouse Anti-GAPDH (1/5000, Fitzgerald 10R-G109A), Goat anti-MCM2 (1/1000, Bethyl A300-122A), Rabbit anti-MCM7 (1/1000, Bethyl A302-584A), and Rabbit anti-MCM4 (1/1000, Bethyl A300-193A). HRP labelled Goat anti-Mouse or Rabbit, or Donkey anti-Goat secondary antibodies were used to visualize protein expression using chemiluminescence substrate (SuperSignal West Dura Extended Duration Substrate, Thermo 34076) on a ChemiDoc system (Biorad). Densitometry analysis was performed with the ImageStudio Lite v5.2.5 (Licor).

### Flow cytometry

For flow cytometry analysis, cells were induced with Doxycycline (500 ng/ml) for 48 hrs, and subsequently treated with Nocodazole or DNA damage inducing agents to test the integrity of the mitotic and G2 checkpoints, respectively, or to test the DNA damage response. At indicated time points, cells were resuspended dropwise in ice-cold 80% ethanol and stored at -20°C until further analysis. All primary stainings were performed overnight at 4°C. Cells were subsequently incubated with FITC-conjugated secondary antibodies (1/50, DAKO) and counterstained with Propidium Iodide (PI) (5 μg/ml) and RNAseA (10 μg/ml) solution to assess DNA content. Data were acquired by flow cytometry on a FACSCalibur station (Becton Dickinson) and analyzed with FlowJo software (v10.0.7.2, FlowJo, LLC).

*Nocodazole challenge*: To test the integrity of the mitotic checkpoint, Doxycycline-induced cells were treated with Nocodazole (12 hrs, 100 ng/ml) to challenge the spindle assembly checkpoint (SAC). Cells were fixed as described above, and the mitotic fraction was assessed by staining for the mitotic histone phosphorylation marks Mouse anti-H3S10P (1/200, Active motif 39636), Rabbit anti-H3S28P (1/200, Abcam ab32388) and Rabbit anti-H3.3S31P (1/250, Abcam ab92628).

*Analysis of G2 checkpoint integrity with Nocodazole block*: To determine the integrity of the G2 checkpoint, asynchronized Doxycycline-induced cells were treated with UVC (10 J/m^2^, UVC500 Crosslinker, Amersham Biosciences Corp.), a 1 hr pulse of Cisplatin (5 μM, Accord), or y-rays (2 Gy, IBL 637 Cesium-137γ-ray machine) to induce DNA damage. Subsequently, progression through mitosis was blocked by Nocodazole treatment (100 ng/ml). Cells were fixed 24 hrs after the induction of DNA damage as described above, and mitotic content was quantified with Mouse anti-H3S10P staining (1/200, Active motif 39636)

*ɣH2AX phosphorylation time curve and cell cycle analysis*: 48 hrs Doxycycline treated cells were UVC irradiated (15 J/m^2^, UVC500 Crosslinker, Amersham Biosciences Corp.) and fixed at 1 and 6 hrs after irradiation in 1% paraformaldehyde, resuspended dropwise in ice-old 80% ethanol and stored at -20°C for at least 24 hrs. DNA damage response was assessed by staining for Rabbit anti-ɣH2AX (1/200, Cell Signaling 9718).

### Statistics

Statistical analysis was done using GraphPad Prism (version 9.1.2). Significance of statistical testing was assessed by two-tailed, (un)paired tests as described in figure legends, and is indicated as follows: ****P < 0.0001, ***P < 0.001, **P < 0.01, and *P < 0.05. ns, not significant. Error bars represent SD (standard deviation).

## Supporting information

S1 Fig H3.3^K27M^ mutant cells have an intact mitotic checkpoint. **(A)** Experimental outline for testing mitotic checkpoint efficiency in H3.3^WT^, H3.3^K27M^, and EV cells. All three cell lines were treated with Doxycycline for 48 hrs to induce FLAG-histone H3.3 overexpression (wild-type or K27M mutant). Cells were subsequently treated with Nocodazole for 12 hrs to induce a mitotic block in early mitosis (prometaphase). Cells were then harvested for flow cytometry analysis. **(B)** Nocodazole or DMSO treated cells were analyzed for H3S10P, H3S28P and H3.3S31P expression to determine the efficiency of histone phosphorylation in mitosis, and the proportion of cells arrested at the mitotic checkpoint using flow cytometry (left panels). Data presented as means ±SD (n = 3 experiments) plotted in bar charts (right panels). *P = 0.0487 and *P = 0.0475 (two-way ANOVA, Tukey correction for multiple comparisons). **(C)** Time spent in mitosis was determined by time lapse imaging form nuclear envelope breakdown to end of cytokinesis. Data represent mean mitotic length ± SD (n = 1 experiment), with a minimum of n = 49 mitotic cells per condition (Mann Whitney non-parametric t-test).
(TIF)

S2 Fig FLAG-immunoprecipitation of synchronized mitotic cells identifies H3.3WT and H3.3K27M protein networks. **(A)** Cells were treated with Doxycycline for 48 hrs and synchronized in mitosis with Nocodazole (8 hrs). The mitotic population was harvested by mitotic shake-off. Wild-type or mutant FLAG-H3.3 were then immunoprecipitated, and the identification of their binding partners was performed by LC-MS/MS and validated by Western blotting. **(B)** A fraction of the input samples was analyzed for mitotic marker H3S10P expression to demonstrate enrichment of the mitotic population using flow cytometry. **(C)** Common histone H3.3WT and H3.3K27M binding partners analyzed in STRING. The illustrated network map was simplified by manual curation for clarity. Dashed circles represent a group of interacting proteins belonging to one biological process. **(D)** GO-term analysis (Biological processes) of common interacting proteins using STRING reveals significant enrichment for translation, splicing and chromosome organization. **(E)** Histone variant binding in H3.3^WT^ and H3.3^K27M^, plotted according to differential spectral counts.
(TIF)

S3 Fig H3.3^K27M^ mutant cells are more sensitive to UVC induced DNA damage. **(A)** Proliferation assays show increased sensitivity of H3.3^K27M^ cells to UVC, but not to ƔRays at 7 days post exposure. Data are represented as means ±SD (n = 2 or 3 experiments). J = joules, Gy = Grey, ns = P>0.05 (two-way ANOVA, Tukey correction for multiple comparisons). **(B)** Flow cytometry analysis reveals ƔH2AX phosphorylation kinetics and dynamics at 1, and 6 hrs following 15 J/m^2^ UVC exposure. Cells were counterstained with Propidium Iodide. **(C)** Quantification of ƔH2AX phosphorylation by flow cytometry. Data are represented as means ±SD (n = 4 experiments), *P = 0.0482 (two-way ANOVA, Tukey correction for multiple comparisons). **(D)** Cell cycle analysis at 24 hrs following 15 J/m^2^ UVC damage by flow cytometry. Representative example of normal cell cycle profiles (untreated, left panels) and UVC treated (right panels) cell cycle profiles. **(E)** Quantification of the proportion of cells in G2/M phase by flow cytometry. Data are represented as means ±SD (n = 3 experiments), *P = 0.0478, **P = 0.0039 (one-way ANOVA, Tukey correction for multiple comparisons). **(F)** Experimental outline for quantification of G2 DNA damage checkpoint activation and efficiency. Following DNA damage (UVC, ƔRays or Cisplatin pulse), cells were blocked in mitosis with Nocodazole to capture the cells that leak through the G2 block. **(G)** Flow cytometry analysis of the mitotic population using H3S10P staining and quantification. Data are represented as means ±SD (n = 3 experiments), ns = P>0.05 (two-way ANOVA, Tukey correction for multiple comparisons). Noco = Nocodazole; CisPt = Cisplatin. **(H)** Quantification of Bloom coated DNA UFBs upon UVC treatment (4 J/m2) or control treatment (no irradiation) of RPE1 cells. Data are represented as means ±SD (n = 3 experiments with > 30 mitoses per condition), *P = 0.0105; **P = 0.0072 and P** = 0.002, (one-way ANOVA, Tukey correction for multiple comparisons).
(TIF)

S1 Table Copy number gains and losses in pediatric high-grade gliomas. Data originally published as Mackay et al, Cancer Cell 2017, DOI: https://doi.org/10.1016/j.ccell.2017.08.017 [4]. Reprinted with permission. Related to Fig 1A.
(XLSX)

S2 Table Spectral counts for proteins that interact with FLAG-H3.3WT and FLAG-H3.3K27M. Proteins that interact with FLAG-H3.3WT and FLAG-H3.3K27M were identified using an immunoprecipitation (IP)/mass spectrometry approach and protein abundance was measured using spectral counts. Related to Fig 2A.
(XLSX)

S3 Table Network map and clustering of common interactors to FLAG-H3.3WT and FLAG-H3.3K27M. Related to S2C and S2D Fig.
(XLSX)

S4 Table Network map and clustering of enriched and specific interactors to FLAG-H3.3K27M. Related to Fig 2C and 2D.
(XLSX)

S5 Table Network map and clustering of enriched and specific interactors to FLAG-H3.3WT. Related to Fig 2E and 2F.
(XLSX)
