# Supplementary figures and images for: The H3.3K27M oncohistone affects replication stress outcome and provokes genomic instability in pediatric glioma
Source: PLoS Genet. 2021 Nov 9;17(11):e1009868. doi: 10.1371/journal.pgen.1009868 (PMC8604337; doi:10.1371/journal.pgen.1009868)

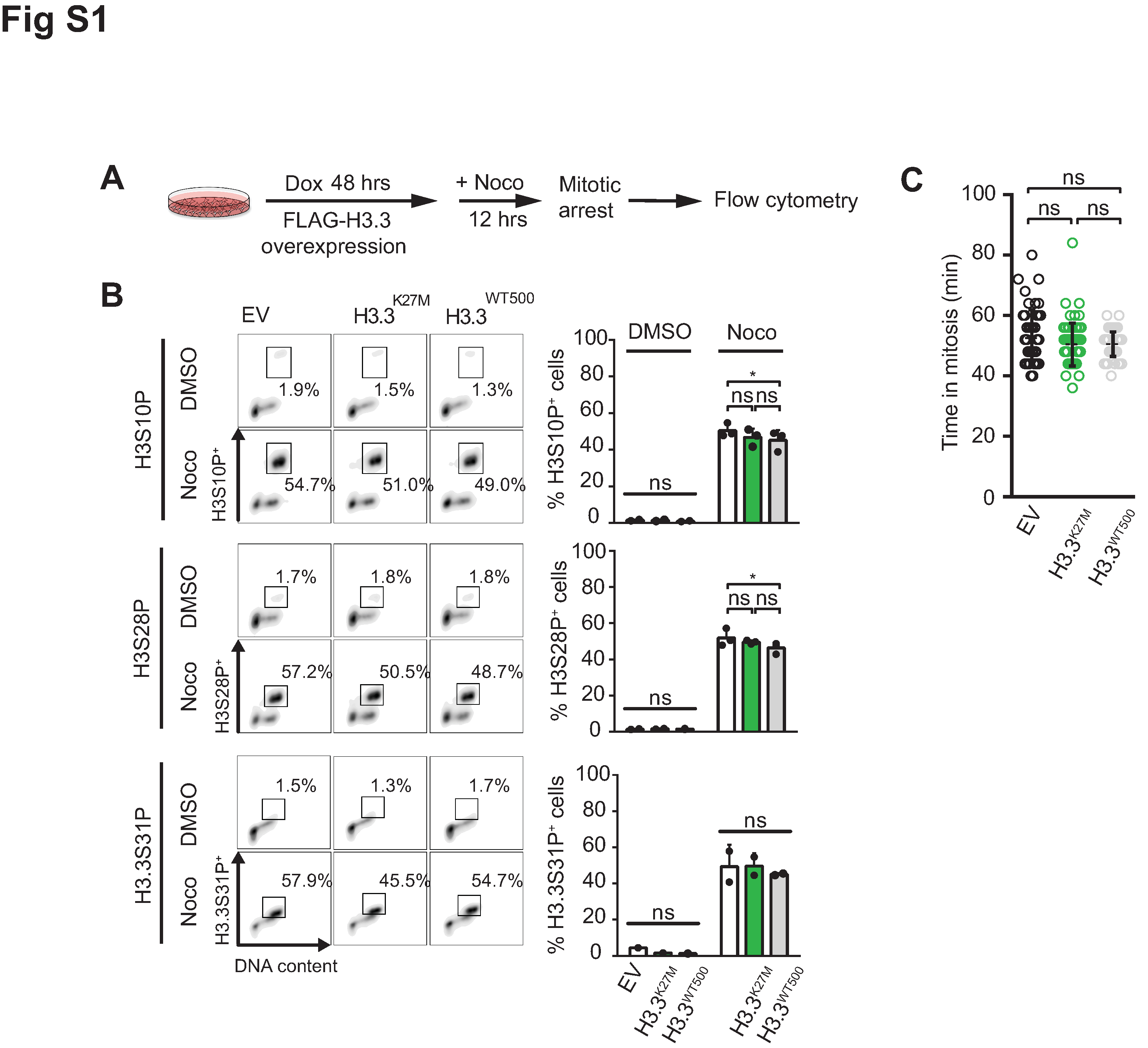

Supplement: S1 Fig — (A) Experimental outline for testing mitotic checkpoint efficiency in H3.3WT, H3.3K27M, and EV cells. All three cell lines were treated with Doxycycline for 48 hrs to induce FLAG-histone H3.3 overexpression (wild-type or K27M mutant). Cells were subsequently treated with Nocodazole for 12 hrs to induce a mitotic block in early mitosis (prometaphase). Cells were then harvested for flow cytometry analysis. (B) Nocodazole or DMSO treated cells were analyzed for H3S10P, H3S28P and H3.3S31P expression to determine the efficiency of histone phosphorylation in mitosis, and the proportion of cells arrested at the mitotic checkpoint using flow cytometry (left panels). Data presented as means ±SD (n = 3 experiments) plotted in bar charts (right panels). *P = 0.0487 and *P = 0.0475 (two-way ANOVA, Tukey correction for multiple comparisons). (C) Time spent in mitosis was determined by time lapse imaging form nuclear envelope breakdown to end of cytokinesis. Data represent mean mitotic length ± SD (n = 1 experiment), with a minimum of n = 49 mitotic cells per condition (Mann Whitney non-parametric t-test). (TIF) [file pgen.1009868.s001.tif]

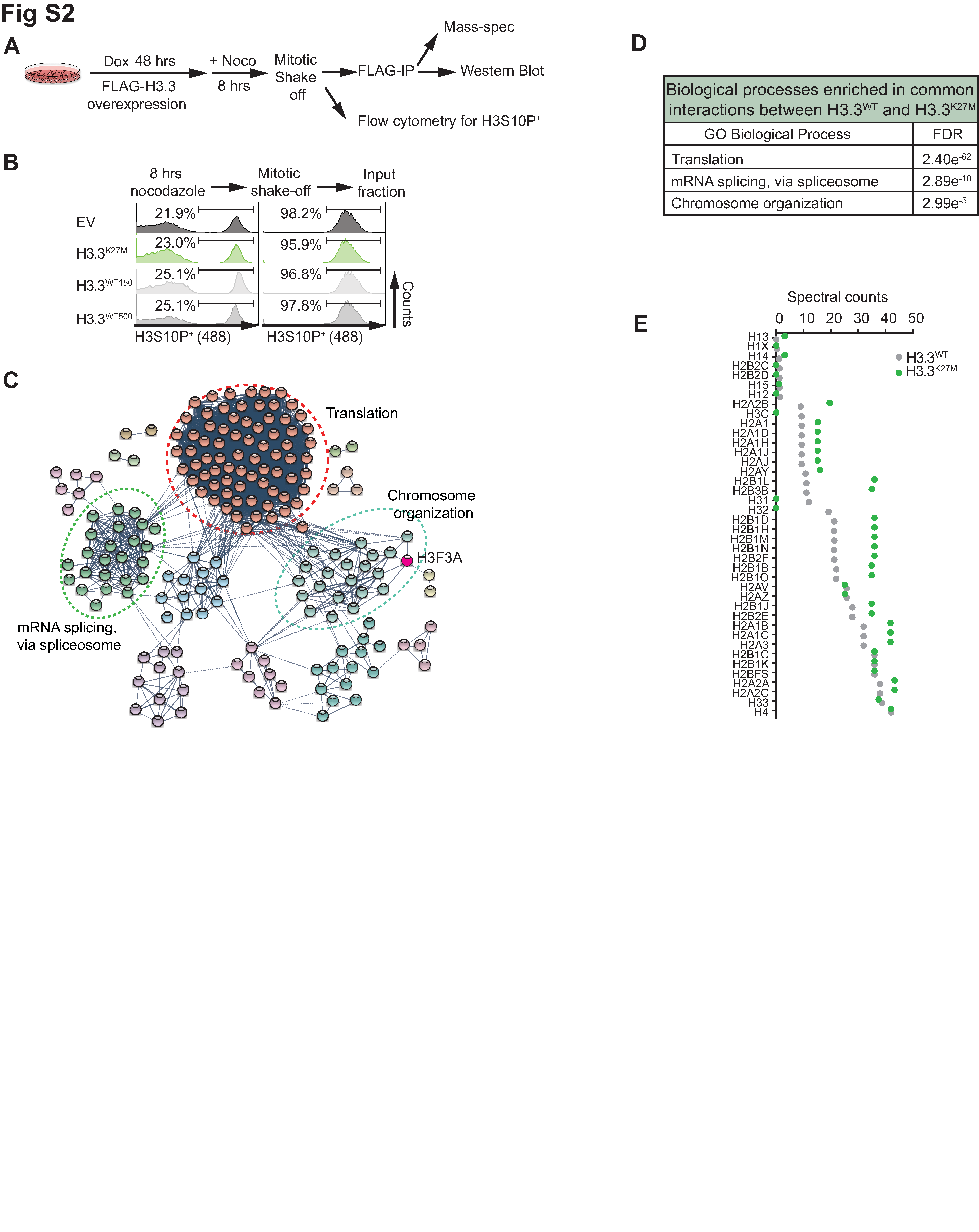

Supplement: S2 Fig — (A) Cells were treated with Doxycycline for 48 hrs and synchronized in mitosis with Nocodazole (8 hrs). The mitotic population was harvested by mitotic shake-off. Wild-type or mutant FLAG-H3.3 were then immunoprecipitated, and the identification of their binding partners was performed by LC-MS/MS and validated by Western blotting. (B) A fraction of the input samples was analyzed for mitotic marker H3S10P expression to demonstrate enrichment of the mitotic population using flow cytometry. (C) Common histone H3.3WT and H3.3K27M binding partners analyzed in STRING. The illustrated network map was simplified by manual curation for clarity. Dashed circles represent a group of interacting proteins belonging to one biological process. (D) GO-term analysis (Biological processes) of common interacting proteins using STRING reveals significant enrichment for translation, splicing and chromosome organization. (E) Histone variant binding in H3.3WT and H3.3K27M, plotted according to differential spectral counts. (TIF) [file pgen.1009868.s002.tif]

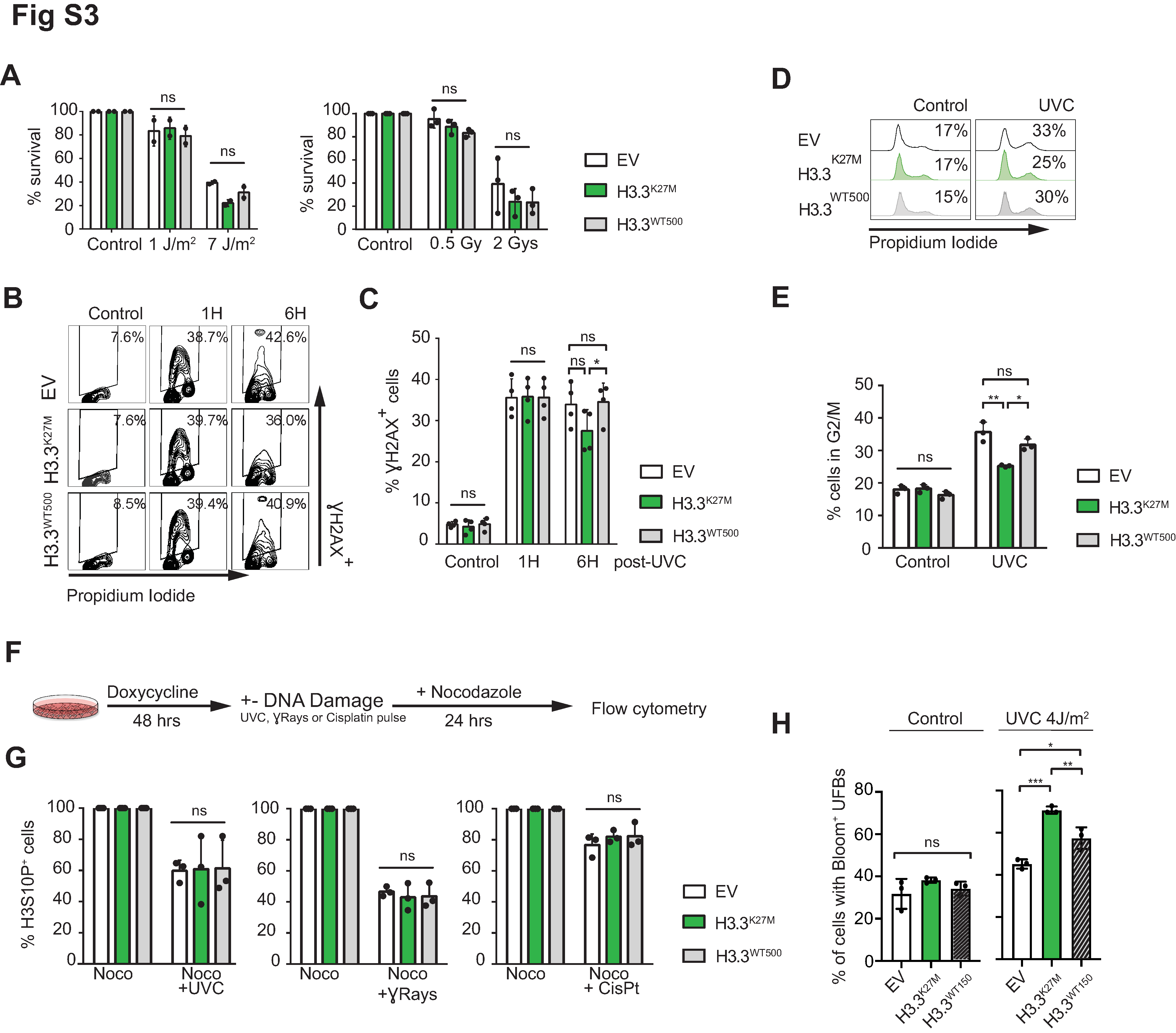

Supplement: S3 Fig — (A) Proliferation assays show increased sensitivity of H3.3K27M cells to UVC, but not to ƔRays at 7 days post exposure. Data are represented as means ±SD (n = 2 or 3 experiments). J = joules, Gy = Grey, ns = P>0.05 (two-way ANOVA, Tukey correction for multiple comparisons). (B) Flow cytometry analysis reveals ƔH2AX phosphorylation kinetics and dynamics at 1, and 6 hrs following 15 J/m2 UVC exposure. Cells were counterstained with Propidium Iodide. (C) Quantification of ƔH2AX phosphorylation by flow cytometry. Data are represented as means ±SD (n = 4 experiments), *P = 0.0482 (two-way ANOVA, Tukey correction for multiple comparisons). (D) Cell cycle analysis at 24 hrs following 15 J/m2 UVC damage by flow cytometry. Representative example of normal cell cycle profiles (untreated, left panels) and UVC treated (right panels) cell cycle profiles. (E) Quantification of the proportion of cells in G2/M phase by flow cytometry. Data are represented as means ±SD (n = 3 experiments), *P = 0.0478, **P = 0.0039 (one-way ANOVA, Tukey correction for multiple comparisons). (F) Experimental outline for quantification of G2 DNA damage checkpoint activation and efficiency. Following DNA damage (UVC, ƔRays or Cisplatin pulse), cells were blocked in mitosis with Nocodazole to capture the cells that leak through the G2 block. (G) Flow cytometry analysis of the mitotic population using H3S10P staining and quantification. Data are represented as means ±SD (n = 3 experiments), ns = P>0.05 (two-way ANOVA, Tukey correction for multiple comparisons). Noco = Nocodazole; CisPt = Cisplatin. (H) Quantification of Bloom coated DNA UFBs upon UVC treatment (4 J/m2) or control treatment (no irradiation) of RPE1 cells. Data are represented as means ±SD (n = 3 experiments with > 30 mitoses per condition), *P = 0.0105; **P = 0.0072 and P** = 0.002, (one-way ANOVA, Tukey correction for multiple comparisons). (TIF) [file pgen.1009868.s003.tif]
